# Ecological Observations Based on Functional Gene Sequencing Are Sensitive to the Amplicon Processing Method

**DOI:** 10.1128/msphere.00324-22

**Published:** 2022-08-08

**Authors:** Fabien Cholet, Agata Lisik, Hélène Agogué, Umer Z. Ijaz, Philippe Pineau, Nicolas Lachaussée, Cindy J. Smith

**Affiliations:** a Infrastructure and Environment, James Watt School of Engineering, University of Glasgow, Glasgow, Scotland; b Université de La Rochelle, CNRS, UMR 7266, LIENSs, La Rochelle, France; c Microbiology, School of Natural Sciences, National University of Ireland, Galway, Ireland; University of California, Davis

**Keywords:** amplicon sequencing, functional genes, nitrogen cycle, amplicon sequence variant, coastal sediments

## Abstract

Until recently, the *de facto* method for short-read-based amplicon reconstruction was a sequence similarity threshold approach (operational taxonomic units [OTUs]). This has changed with the amplicon sequence variant (ASV) method where distributions are fitted to abundance profiles of individual genes using a noise-error model. While OTU-based approaches are still useful for 16S rRNA/18S rRNA genes, where thresholds of 97% to 99% are used, their use for functional genes is still debatable as there is no consensus on clustering thresholds. Here, we compare OTU- and ASV-based reconstruction approaches and taxonomy assignment methods, the naive Bayesian classifier (NBC) and Bayesian lowest common ancestor (BLCA) algorithm, using a functional gene data set from the microbial nitrogen-cycling community in the Brouage mudflat (France). A range of OTU similarity thresholds and ASVs were used to compare *amoA* (ammonia-oxidizing archaea [AOA] and ammonia-oxidizing bacteria [AOB]), *nxrB*, *nirS*, *nirK*, and *nrfA* communities between differing sedimentary structures. Significant effects of the sedimentary structure on weighted UniFrac (WUniFrac) distances were observed for AOA *amoA* when using ASVs, an OTU at a threshold of 97% sequence identity (OTU-97%), and OTU-85%; AOB *amoA* when using OTU-85%; and *nirS* when using ASV, OTU-90%, and OTU-85%. For AOB *amoA*, significant effects of the sedimentary structures on UniFrac distances were observed when using OTU-97% but not ASVs, and the inverse was found for *nrfA*. Interestingly, conclusions drawn for *nirK* and *nxrB* were consistent between amplicon reconstruction methods. We also show that when the sequences in the reference database are related to the environment in question, the BLCA algorithm leads to more phylogenetically relevant classifications. However, when the reference database contains sequences more dissimilar to the ones retrieved, the NBC obtains more information.

**IMPORTANCE** Several analysis pipelines are available to microbial ecologists to process amplicon sequencing data, yet to date, there is no consensus as to the most appropriate method, and it becomes more difficult for genes that encode a specific function (functional genes). Standardized approaches need to be adopted to increase the reliability and reproducibility of environmental amplicon-sequencing-based data sets. In this paper, we argue that the recently developed ASV approach offers a better opportunity to achieve such standardization than OTUs for functional genes. We also propose a comprehensive framework for quality filtering of the sequencing reads based on protein sequence verification.

## INTRODUCTION

PCR combined with high-throughput sequencing (HTS) has revolutionized our understanding of microbial ecology (1). Amplicon sequencing of the *16S rRNA* gene as a molecular marker for diversity is now routine (2). This approach can also be applied to genes and/or transcripts encoding enzymes for specific functions (functional genes). Functional genes involved in cell division and maintenance (housekeeping genes) can be used as an alternative to the *16S rRNA* gene for diversity estimation (3, 4) to circumvent the issue of intragenomic variation in the 16S rRNA gene (5). Biogeochemical cycles can also be targeted via key functional genes involved in the processes of interest, e.g., the nitrogen cycle (6–11), the sulfur cycle (12, 13), and the methane cycle (14). The same is true for bioremediation (15, 16) and antibiotic resistance (17), to mention but a few possibilities. By targeting functional genes, we can start to unravel the functional potential of microbial communities, and if transcripts are targeted, actively transcribing organisms are revealed, a step closer to identifying the organisms driving the target processes.

Irrespective of the gene target, before hypothesis testing and ecological meaning can be inferred from amplicon data sets, the sequences first have to be grouped into taxonomically meaningful “units” to allow downstream analysis. There are two approaches for grouping amplicon data for downstream analysis: operational taxonomic units (OTUs) and amplicon sequence variants (ASVs). OTUs group sequences into a consensus sequence (the OTU) at a defined sequence similarity threshold. For the *16S rRNA* gene, a threshold of 97% sequence identity is generally used to define OTUs to the species level, although this value has been challenged (18). As functional genes may have been subject to significant horizontal gene transfer and may be present in poly- or monophyletic groups, the relationship between percent identity and taxonomic delimitation is not clear and is often unknown. As a result, in the literature, different similarity thresholds have been used to construct OTUs for the same functional gene target (Table 1), with an OTU at a threshold of 97% sequence identity (OTU-97%) being widely used, often without a clear rationale. This variation in OTU selection criteria makes it difficult to select a meaningful value and creates limitations when comparing data among studies. This is important as uncertainties in selecting the appropriate taxonomic cutoff could lead to different interpretations of findings underpinning our understanding of larger-scale ecological processes and mechanisms structuring microbial communities and their activities (19). For the *16S rRNA* gene, the choice of OTU similarity threshold used can significantly influence microbial diversity patterns (20, 21), and we hypothesize that this is also true for functional genes. In fact, some authors suggested that OTU similarity thresholds should be adjusted depending on the clustering algorithm and data complexity when phylogenetically divergent groups are present within the same community. This is because a single threshold for species delimitation is often not relevant due to the variable evolutionary rates of the *16S rRNA* gene across lineages (22, 23). Indeed, the use of strict thresholds has been shown to result in phylogenetically inconsistent (para- or polyphyletic) OTUs (24, 25).

**TABLE 1:** Range of OTU similarity thresholds currently used in the literature for nitrogen-cycling genes to construct OTUs

Gene	Primer pair	Reference(s) reporting similarity threshold of:					
		97%	95%	90%	85%	83%	82%
AOB *amoA* (bacterial ammonia monooxygenase)	BacamoA-1F/BacamoA-2R	7, 8, 72–76	77 – 79	80	81, 82		
AOA *amoA* (archaeal ammonia monooxygenase)	Arch-amoWAF/Arch-amoWAR	7, 8, 73, 74, 76, 83, 84	77, 79, 81	85	80 – 82		
*nirK* (nitrate reductase)	nirKFlaCu/nirKR3Cu	86 – 88	79	89		90	
*nirS* (nitrate reductase)	nirS1F/nirS3R	87, 88	91	89			90
*nrfA* (nitrate reductase to ammonia)	nrfAF2aw/nrfAR1	92					
*nxrB* (nitrite oxidase)	nxrB169f/nxrB638r	93					

After selection of the sequence similarity threshold, further choices need to be made. OTU clustering can be done using a closed-reference approach (22, 26) or by *de novo* assembly (22, 26). With the latter method, reads are clustered into OTUs without comparison to a preexisting database. This helps with the discovery of novel OTUs that are dissimilar to known sequences. However, the absence of comparison to known references makes *de novo* OTUs dataset-dependent, meaning that *de novo* OTUs from two different studies cannot be directly compared. On the other hand, the former approach makes it easier to compare OTUs between studies but limits the possibilities of discovering new sequences (26). The open-reference OTU-picking method combines the closed-reference and *de novo* methods: reads are clustered against a reference database, and those that do not match reference sequences are clustered *de novo*. Even though this seems a good compromise between closed-reference and *de novo* methods, problems such as inflated richness and exaggerated between-sample diversity can still occur (27).

With the ASV approach (26), an error model is generated for the sequencing run, and reads are clustered in order to map this error model. The two most commonly used pipelines for ASV reconstruction are DADA2 (28), in which both forward and reverse reads are denoised and merged, and Deblur (29), in which forward reads can be used on their own to reconstruct ASVs. This is advantageous when amplicons are too long to be merged properly or when the quality of the reverse reads is poor (30). The ASV approach is appealing as it no longer groups amplicons based on a consensus sequence but instead resolves sequences with as little as a single nucleotide variation. Consequently, the ASV method does not require reference databases and is able to detect new sequences, and ASVs from different data sets can be directly compared (26). The ASV method has been compared to the OTU approach using phylogenetic markers (the 16S rRNA gene, the 18S rRNA gene, and the fungal internal transcribed spacer [ITS] region), and overall, the results indicate better accuracy (31–33) and sensitivity (34) of the former when tested against mock communities. The impact that this has on large-scale ecological patterns still needs to be fully understood as some research suggests that the biological conclusions drawn using either method, based on phylogenetic markers, are largely consistent (32, 35), while others suggest that HTS processing method (OTU versus ASV) can affect the interpretation of differentially abundant taxa between treatments (36). Nonetheless, as the use of ASVs does not rely on a user-defined threshold that may not hold biological meaning, this approach should increase the phylogenetic resolution of functional genes and, importantly, facilitate comparison of data among studies, as they should be able to segregate sequences on as little as one nucleotide variant. Consequently, ASVs are a promising approach for functional gene amplicon studies (13). However, how the use of ASVs versus OTUs impacts ecological interpretations based on functional genes is unclear.

After selecting the appropriate amplicon reconstruction method (ASVs or OTUs), the next immediate challenge is assigning taxonomy by matching against a reference database. For the *16Sr RNA* gene, several curated databases are available (e.g., Silva, GreenGenes, and Midas) and are routinely used. For most functional genes, no such databases are available, and the reference sequences used to assign taxonomy often vary among studies. The most popular approaches for assigning taxonomy rely on the pattern recognition of overlapping “words” of length k (generally k = 8), called k-mers. The frequency of matching k-mers between the query and reference sequences is used as a measure of sequence similarity: a higher frequency of shared k-mers indicates higher similarity between the query and reference sequences. This approach is fast, objective, and not limited by the uncertainties associated with methods based on evolutionary models and alignments (37, 38). This approach is usually implemented as a classifier, such as the commonly used naive Bayesian classifier (NBC). One main limitation of this approach is that it uses the assumption that the actual position of the k-mers in the sequence is not important, whereas in reality, two sequences with the same k-mers but in different orders are different. Furthermore, the optimal choice of length for the k-mer might vary depending on the target gene or the region within the same gene (39). Another approach is the Bayesian lowest common ancestor (BLCA) algorithm (39), where the query sequence is subjected to a BLAST search against a reference database(s) and significant “hits” are recorded. The taxonomy of the query sequence is assigned as the lowest common ancestor between these hits. For example, if a query sequence has significant matches to two Nitrosomonas europaea reference sequences, the query sequence will be assigned to the species Nitrosomonas europaea as it is the lowest common ancestor between the two hits. However, if a query sequence has two different hits, Nitrosomonas europaea and Nitrosomonas oligotropha, the query sequence will be assigned to the genus *Nitrosomonas*. By considering hit results from multiple databases, the BLCA approach is able to provide probabilistic-based confidence values at each taxonomic level of this assignment. Previously, the BLCA method was shown to provide better species-level resolution than the NBC for *16S rRNA* gene sequences (39, 40). How they compare for functional genes has yet to be determined.

The aim of this study is to compare the effects of amplicon reconstruction approaches, OTUs (at a range of sequence similarity values) versus ASVs, and taxonomic assignment methods, NBC versus BLCA, on a suite of functional genes. We do this to determine if diversity measures and subsequent ecological interpretations are affected by these choices. We hypothesize that both alpha and beta diversity measures will differ depending on the amplicon-processing methods used. To do this, we examine the nitrogen cycle in marine sediments of Marennes-Oléron Bay on the French Atlantic coast. The middle part of the bay, the Brouage mudflat, is characterized by the presence of flow-parallel sediment structures consisting of crests (ridges) and troughs (runnels). These side-by-side physical structures have been shown to significantly affect the nitrification rates (higher in runnels) (41, 42). We ask if the physical structure of the ridges and runnels results in differences in the diversity of the nitrogen-cycling community present. Different pathways of the nitrogen cycle are targeted via genes encoding key enzymes. Specifically, nitrification, the oxidation of ammonia to nitrate via nitrite, is targeted via subunit A of ammonia monooxygenase (*amoA*) and the beta subunit of nitrite oxidoreductase (*nxrB*); denitrification, the sequential reduction of nitrate to dinitrogen gas, is targeted via the nitrite reductase genes *nirS* and *nirK*; and dissimilatory nitrate reduction to ammonia (DNRA) is targeted via the cytochrome *c* nitrite reductase gene *nrfA*.

## RESULTS

### Conversion of raw reads to OTUs/ASVs.

For all genes except *nrfA* and *amoA* from ammonia-oxidizing archaea (AOA *amoA*), there was a high percentage (~70% to ~95%) of reads converted from raw reads to OTUs/ASVs. The use of OTUs generally resulted in a higher percentage of reads being retained for *amoA* from ammonia-oxidizing bacteria (AOB *amoA*) (80.57% to 84.74%) than the use of ASVs (79.61%). An opposite trend was observed for *nxrB* (87.03% for ASVs versus 77.55% for OTUs), *nirK* (95.33% for ASVs versus 86.41% to 93.45% for OTUs), and *nirS* (90.92% for ASVs versus 77.76% to 78.98% for OTUs). For AOA *amoA* and *nrfA*, low percentages of reads were retained when using OTUs (32.86% for *nrfA* and 14.50% to 30.26% for AOA *amoA*), whereas the ASV method allowed a good conversion of raw reads (62.75% for *nrfA* and 94.43% for AOA *amoA*) (Table 2).

**TABLE 2:** Read loss during the processing of HTS data using OTU and ASV pipelines

Gene and pipeline	No. of reads for OTU/ASV processing^*a*^							Quality filtering (AA sequences)				
	Raw reads	Quality filtering	Correction	Merging	Chimeras	Final conversion to ASV/OTU	Unique ASVs/OTUs	No. of filtered reads	% raw reads	% processed reads	% wrong ASVs/OTUs	Final no. of unique ASVs/OTUs
AOB *amoA*												
ASV	1,086,576	1,086,576	NA	993,017	98,787	894,230	910	864,996	79.61	96.73	57.80	384
OTU-97%	1,086,576	1,084,818	1,084,051	1,072,646	12,620	907,973	274	875,443	80.57	96.42	79.20	57
OTU-95%	1,086,576				6,565	938,258	205	895,785	82.44	95.47	83.90	33
OTU-90%	1,086,576				22	974,070	166	910,044	83.75	93.43	90.96	15
OTU-85%	1,086,576				2	994,878	151	920,739	84.74	92.55	94.70	8
AOA *amoA*												
ASV	1,714,971	1,713,630	NA	1,660,869	36,563	1,624,306	596	1,619,524	94.43	99.71	4.53	569
OTU-97%	1,714,971	1,707,358	1,705,368	591,774	7	251,981	137	248,737	14.50	98.71	18.98	111
OTU-95%	1,714,971				0	322,959	64	314,603	18.34	97.41	21.88	50
OTU-90%	1,714,971				28	414,551	21	410,824	23.96	99.10	28.57	15
OTU-85%	1,714,971				0	522,952	12	518,934	30.26	99.23	50.00	6
*nxrB*												
ASV	297,508	297,404	NA	285,919	24,575	285,919	861	258,935	87.03	90.56	5.23	816
OTU-97%	297,508	297,295	296,968	296,494	146	249,994	372	230,712	77.55	92.29	10.48	333
*nirK*												
ASV	1,317,256	1,316,137	NA	1,274,893	7,957	1,266,936	2,331	1,258,340	95.53	99.32	2.27	2,278
OTU-97%	1,317,256	1,305,520	1,302,639	1,150,918	1,842	1,169,122	1,074	1,138,188	86.41	97.35	5.77	1,012
OTU-95%	1,317,256				1,693	1,192,377	918	1,178,126	89.44	98.80	5.01	872
OTU-90%	1,317,256				62	1,215,020	701	1,210,577	91.90	99.63	5.42	663
OTU-83%	1,317,256				2	1,235,483	330	1,230,920	93.45	99.63	10.30	296
*nirS*												
ASV	1,410,146	1,409,380	NA	1,326,808	42,336	1,284,472	2,190	1,282,047	90.92	99.81	5.57	2,068
OTU-97%	1,410,146	1,404,968	1,404,097	1,215,878	42	967,658	248	960,374	68.10	99.25	15.32	210
OTU-95%	1,410,146				169	1,052,876	204	1,045,334	74.13	99.28	14.71	174
OTU-90%	1,410,146				0	1,124,335	155	1,113,700	78.98	99.05	14.19	133
OTU-82%	1,410,146				0	1,124,622	63	1,096,597	77.76	97.51	25.40	47
*nrfA*												
ASV	1,829,948	1,829,295	NA	1,603,920	444,687	1,159,233	7,520	1,148,346	62.75	99.06	1.41	7,414
OTU-97%	1,829,948	1,829,382	1,828,735	1,219,228	8	614,215	117	601,388	32.86	97.91	11.97	103

a NA, not applicable.

### OTU/ASV quality check.

**FIG 1:**
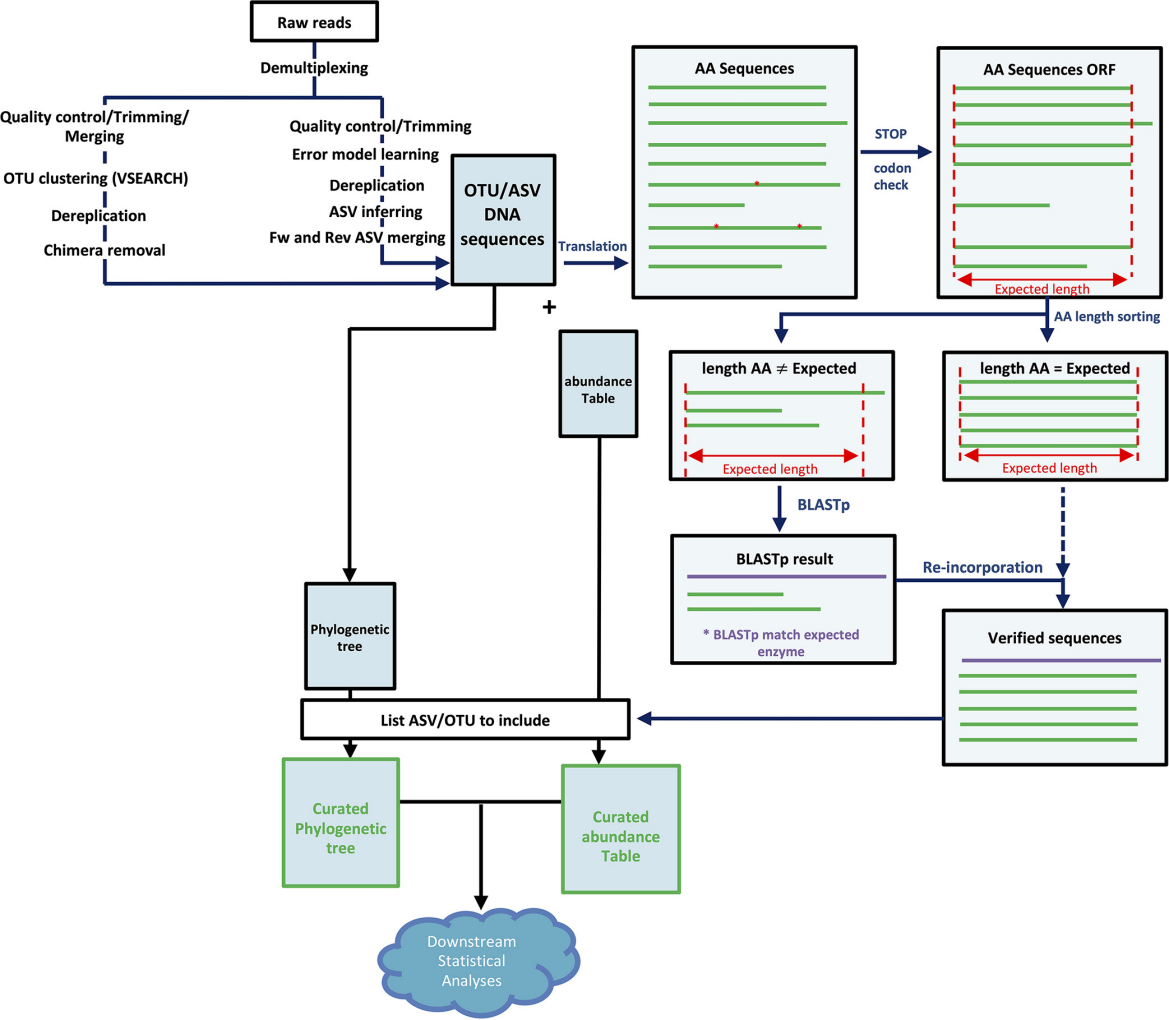
OTU/ASV quality check workflow (example of the bacterial *amoA* gene). Red asterisks indicate stop codons. Fw, forward; Rev, reverse; ORF, open reading frame.

A quality check based on translated OTU/ASV sequences was performed to ensure that only high-quality reads were retained for downstream analyses. First, amino acid (AA) sequences containing stop codons were deleted. Next, AA sequences were sorted depending on their sizes. Those shorter or longer than the expected size were subjected to a BLAST analysis using BLASTp and were retained only if they matched the expected enzyme (Fig. 1). A high number of sequences were found to either contain stop codons or not translate to the correct protein (e.g., for AOB *amoA*, 57.8% to 94.70% of the sequences did not pass the quality-filtering step). For all genes tested, even though the absolute number of error-prone sequences increased, their proportion decreased when using ASVs compared to OTUs and increased as the percent similarity decreased for OTU construction (Table 2). These error-prone sequences, despite being numerous, represented only a small fraction of the total abundance. Indeed, between ~90% and ~99% of the reads that passed the processing steps were retained after the quality-filtering step.

As shown in Fig. S1 in the supplemental material, there was an overlap between the sizes of the correct and error-prone AA sequences. A simple gating system based on protein size could therefore not be used to automatically remove the error-prone sequences. A manual check based on a BLAST analysis of AA sequences that are shorter or longer than the expected length therefore remains the best option for verification. The only exception was for AOB *amoA* when using ASVs, where only the AA sequences with exactly the expected length were found to be correct proteins.

10.1128/msphere.00324-22.2 FIG S1 Size distribution of representative ASVs/OTUs. The numbers of representative ASVs/OTUs (left *y* axis) and their abundances (right *y* axis) are plotted against their sizes (*x* axis) (from 0 to 600 bp with 10-bp intervals). For each interval, the color of the bar represents the percentage of sequences that match the expected enzyme. Download FIG S1, PDF file, 0.3 MB. Copyright © 2022 Cholet et al. 2022 Cholet et al. https://creativecommons.org/licenses/by/4.0/ This content is distributed under the terms of the Creative Commons Attribution 4.0 International license.

### Effects of OTU thresholds and ASV selection on alpha diversity and sequence coverage.

**FIG 2:**
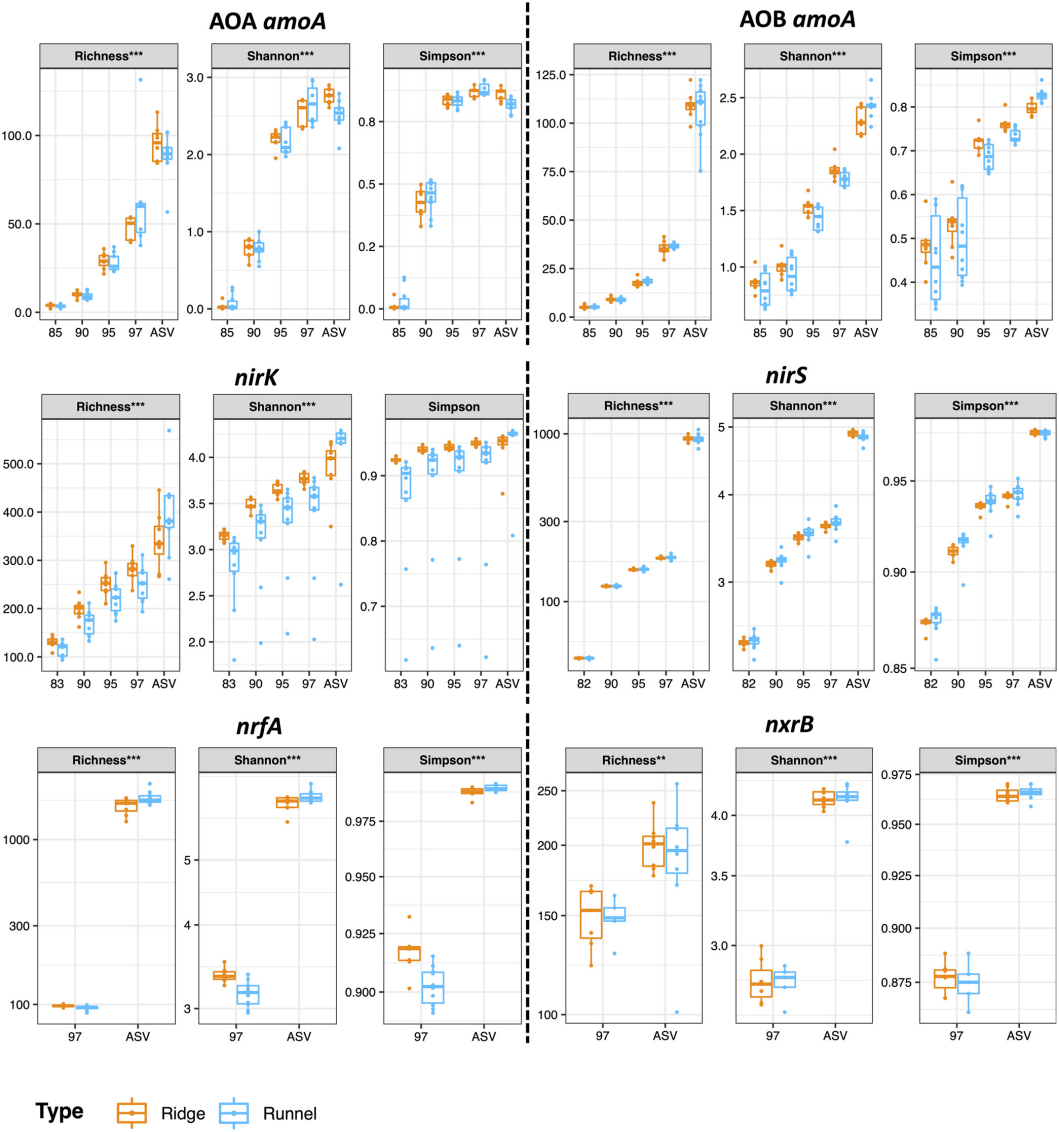
Effects of different OTU sequence similarity thresholds versus ASVs on alpha diversity results. Results of analysis of variance (ANOVA) for the effect of the clustering method on richness, Simpson, and Shannon indices are reported at the top of each plot for each gene. *, *P* < 0.05; **, 0.01 > *P* > 0.001; ***, *P* < 0.001.

**(i) Alpha diversity indices.** Richness, Simpson, and Shannon indices were calculated based on the rarefied abundance tables. To allow comparison between amplicon-processing methods, abundance tables were rarefied to 10,000 reads. Samples that contained fewer than 10,000 reads were not included in the analysis. In general, an increase in the percent identity used to generate OTUs resulted in an increase in the values of alpha diversity indices (Fig. 2). A similar increase was generally observed when using ASVs instead of OTUs. The increases in alpha diversity indices between OTUs and ASVs were particularly strong for *nirS* and *nrfA* (all indices) and AOB *amoA* (richness). For AOA *amoA*, there were slight decreases in Shannon and Simpson indices from OTU-97% to ASVs for runnel samples. Interestingly, the use of OTUs (97%) and ASVs could lead to different interpretations as to which of the two sedimentary structures was the most diverse. When using OTU-97%, ridges had higher Shannon (*P* < 0.05) and Simpson (0.01 < *P* < 0.05) index values than runnels, and the inverse was found when using ASVs (0.01 < *P* < 0.05 for Shannon and *P* < 0.001 for Simpson). A similar trend was observed for *nrfA*, with higher richness (0.01 < *P* < 0.05), Shannon (*P* < 0.001), and Simpson (0.01 < *P* < 0.05) values in ridges than in runnels when using OTU-97% and higher richness (*P* < 0.05), Shannon (*P* < 0.05), and Simpson (*P* < 0.05) values in runnels when using ASVs (Fig. 2) (Table S4).

**FIG 3:**
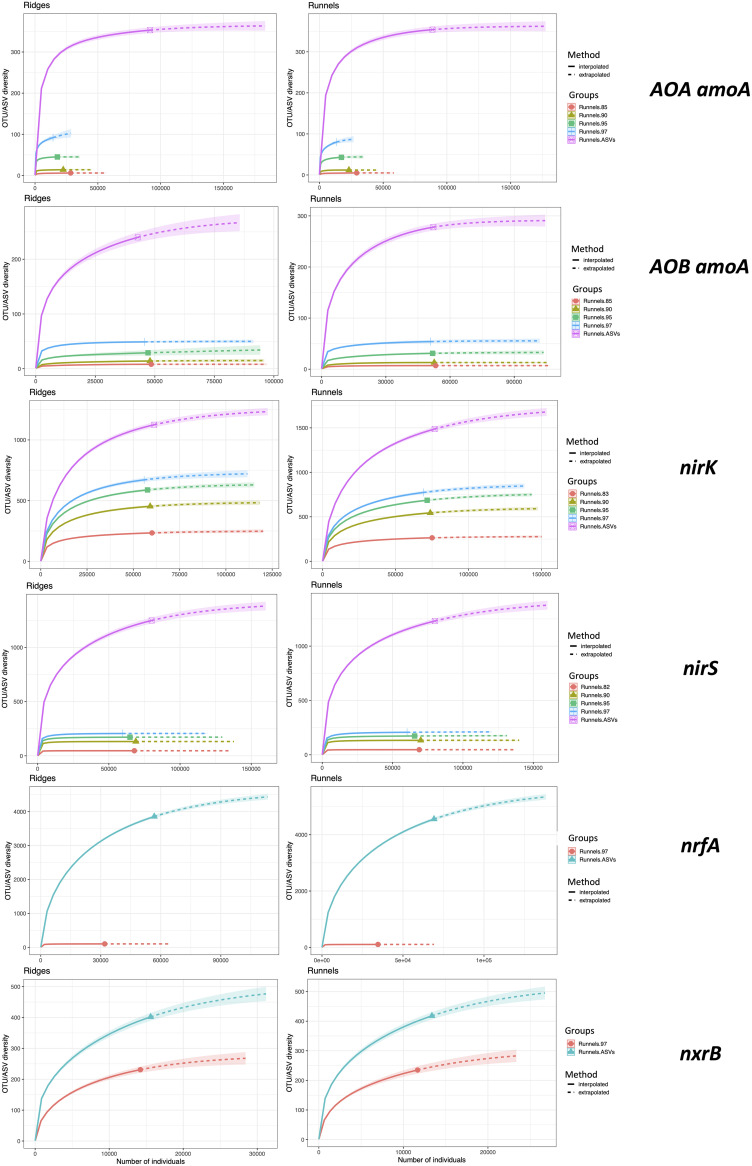
Rarefaction curves in ridges and runnels using different OTU sequence similarity thresholds and ASVs.

**(ii) Rarefaction curves.** To determine if the sequencing effort had been sufficient to capture the full OTU/ASV diversity, rarefaction curves were drawn for all genes using the OTU and ASV abundance tables. When sequencing reads were clustered using the OTU approach, the rarefaction curves generally reached a quasiplateau phase, indicating that the observed richness was close to its maximum theoretical value. For *nirK* and *nxrB*, this plateau phase was not reached, indicating that more OTUs would have been recovered with greater sequencing depth. Rarefaction curves obtained from ASV abundance tables were generally farther from reaching the plateau than curves obtained using the OTU approach (Fig. 3).

### Effect of OTU thresholds and ASVs on beta diversity.

**FIG 4:**
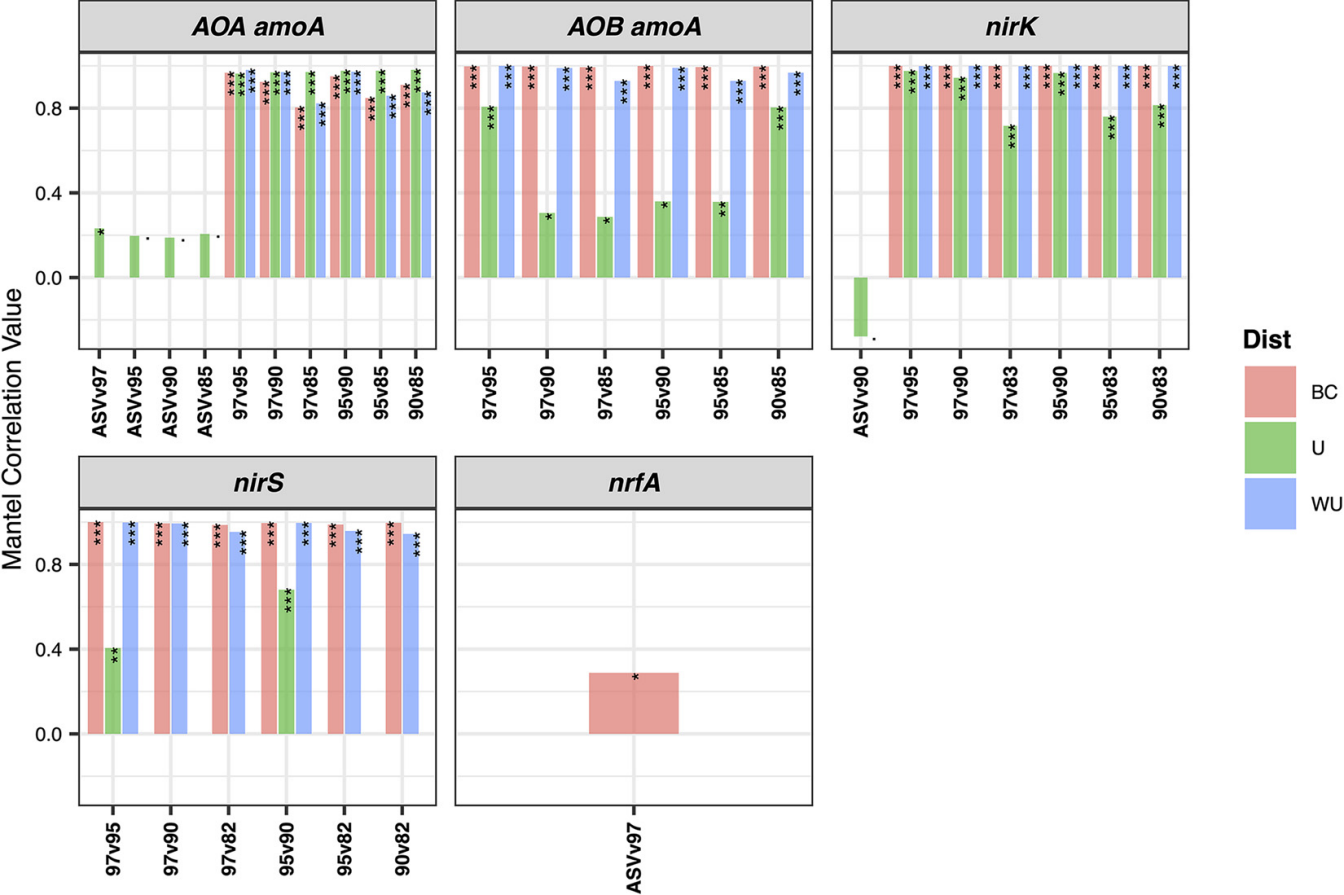
Effects of different OTU sequence similarity thresholds or ASV amplicon reconstruction approaches on phylogenetic distances. Distance matrices were calculated using Bray-Curtis (BC), UniFrac (U), and weighted UniFrac (WU) metrics at different OTU similarity thresholds and ASVs for each gene. Pairwise correlations between matrices, as indicated on the *x* axis, were calculated using a Mantel test. The *P* values of the tests are reported at the top of the bar plots. ·, 0.05 < *P* < 0.1; *, *P* < 0.05; **, 0.01 > *P* > 0.001; ***, *P* < 0.001.

**(i) Dissimilatory distances between samples.** To determine the effect of the amplicon data analysis method on the dissimilarity distance between samples, Mantel correlations were calculated among distance matrices obtained using Bray-Curtis (BC), UniFrac (U), and weighted UniFrac (WUniFrac) metrics. A strong effect of the approach used is seen, as reflected by Mantel correlations different from a value of 1. For all genes tested, low correlations between distance matrices obtained with ASVs versus OTUs were observed (~0.2 or lower [*P* > 0.5]). In contrast, the correlations between distance matrices obtained with OTUs at different similarity thresholds were generally high (>0.8 [*P* < 0.01]), except for the UniFrac distance matrices for AOB *amoA* and *nirS*, where lower correlation values were generally observed (Fig. 4).

**FIG 5:**
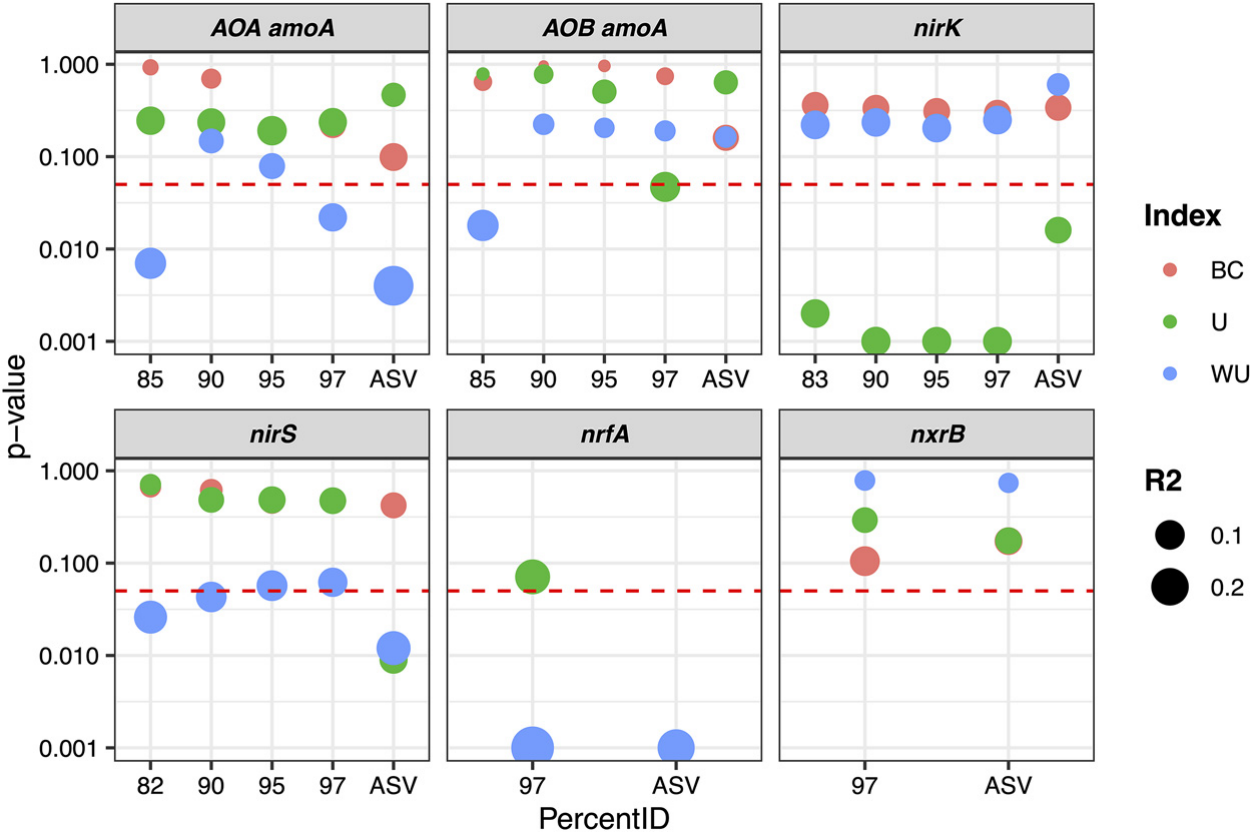
Effect of the amplicon reconstruction method on beta diversity results. The reported *P* values show the effect of the ridge/runnel structure on the community composition determined by the sequencing of six different nitrogen cycle genes using three different dissimilarity distances, indicated by the color of the symbols, as explained in the key. The horizontal dashed red line represents the *P* value threshold for significance (0.05). The size of the points represents the *R*^2^ value, i.e., the percentage of variance in the Bray-Curtis (BC), UniFrac (U), and weighted UniFrac (WU) metrics explained by the ridge/runnel structures.

**(ii) Differences in communities between ridges and runnels.** To determine whether the method used to process sequencing reads could significantly affect the beta diversity results, Bray-Curtis, UniFrac, and WUniFrac distances between communities in the two sedimentary structures (ridges and runnels) were calculated, and their significance was tested using permutational multivariate analysis of variance (PERMANOVA). The effect of the clustering method was different depending on the gene of interest (Fig. 5). For AOA *amoA*, significant differences in WUniFrac distances between ridges and runnels were seen when reads were clustered using the ASVs or OTUs at 97% and 85% similarity thresholds but not when using OTUs at 95% and 90% similarity thresholds. For *nirS*, significant differences were observed for WUniFrac when using ASVs, OTU-82%, and OTU-90% but not when using OTU-95% and OTU-97%. When considering UniFrac distances, significant differences were observed for *nrfA* and *nirS* communities when using ASVs but not when using OTU-97%, and the inverse was found for AOB *amoA*. For *nirK* and *nxrB*, the different clustering methods were more consistent, with no significant effect of the data analysis pipeline on beta diversity (Fig. 5). To summarize, the clustering method used for amplicons had a significant effect on beta diversity for some genes, while other were less affected.

To better understand the effect of the HTS data processing method on the phylogeny of representative sequences, phylogenetic trees were drawn using representative OTUs/ASVs along with full-length or quasi-full-length sequences downloaded from the NCBI or Fungene database. Tree dissimilarities increased as the number of representative sequences in the trees increased following natural logarithm regression [Robinson-Foulds distances, RF.distance = *a* × ln(number of sequences) + *b*], indicating a strong effect of the HTS data processing method on the phylogeny of representative sequences (Fig. S2).

10.1128/msphere.00324-22.3 FIG S2 Comparison of phylogenetic trees. For each gene, phylogenetic trees were drawn using the most abundant OTUs/ASVs. At each iteration, one additional sequence was added, and the normalized Robinson-Foulds metric was computed. Download FIG S2, PDF file, 0.8 MB. Copyright © 2022 Cholet et al. 2022 Cholet et al. https://creativecommons.org/licenses/by/4.0/ This content is distributed under the terms of the Creative Commons Attribution 4.0 International license.

### Effects of OTU thresholds and ASVs on canonical analyses.

Canonical analyses, e.g., canonical correspondence analysis (CCA), are useful approaches to analyze, detect, and visualize interactions between microbial communities and environmental parameters. CCA measures the association between an explanatory table (the physicochemical parameters) and a response table (the abundance table). Previously, it was shown that, in some cases, the amplicon reconstruction method affects the composition of the abundance tables, as revealed by Mantel correlations between Bray-Curtis distance matrices obtained from ASVs or OTUs at different similarity thresholds of <1 (Fig. 4). These changes in the abundance tables can be expected to also change the results of CCA. To test this, CCA was done using the abundance tables obtained from the different amplicon reconstruction methods (range of OTU sequence similarity thresholds and ASVs) using the same physicochemical data table. As shown in Table 3, the choice of amplicon processing method strongly impacted the results, except for *nxrB*, where no significant drivers were found regardless of the method used.

**TABLE 3:** Canonical correspondence analysis^*a*^

Pipeline	Significance								
	NH_3_	NO_2_^−^	NO_3_^−^	ChlA	pH	SGS	TOC	DOC	TDN
AOB ASV				·					
AOB 97					·	·			
AOB 95			·	·	*	*			
AOB 90						*			
AOB 85						·			

AOA ASV						*			
AOA 97	*			*					
AOA 95						*			
AOA 90						**			
AOA 85						*			

*nirK* ASV							*		
*nirK* 97						*	*		
*nirK* 95						·	*		
*nirK* 90							·		
*nirK* 83			·			**	**	·	

*nirS* ASV	***								*
*nirS* 97									
*nirS* 95									
*nirS* 90				·					
*nirS* 82				*					

*nxrB* ASV									
*nxrB* 97									

*nrfA* ASV	***								
*nrfA* 97	·		*	*	·				

a Empty cells indicate that the physicochemical parameter is not a driver of the community. ·, 0.1 > *P* > 0.05; *, *P* < 0.05; **, 0.01 > *P* > 0.001; ***, *P* < 0.001. ChlA, chlorophyll *a*; SGS, sediment grain size (percent <63 μm); TOC, total organic carbon; DOC, dissolved organic carbon; TDN, total dissolved nitrogen; Pipeline' indicates the target gene and the HTS method. e.g. AOB 97, AOB OTU at a threshold of 97% sequence identity.

For ammonia oxidizers, when using ASVs, chlorophyll *a* was a weak driver (0.1 < *P* < 0.05) of AOB communities, and sediment grain size (SGS) was a significant driver of AOA. When using OTUs, ammonia and chlorophyll *a* became significant drivers of AOB at a 97% similarity threshold, while SGS became a significant driver for lower similarity thresholds. For AOA, pH and SGS were significant drivers at a 95% similarity threshold. SGS remained significant at 90%, while pH did not.

For the nitrite-oxidizing community, again, differences were seen depending on the amplicon reconstruction approach. Total organic carbon (TOC) was found to be a driver for *nirK*, except for OTU-90%, while SGS was a significant driver only when using OTU-97% and OTU-83%. For *nirS*, ammonia was a strong driver (*P* < 0.01) only when using ASVs. Total dissolved nitrogen (TDN) and chlorophyll *a* were significant drivers only when using ASVs and OTU-82%, respectively. For *nrfA*, ammonia was also a strong driver (*P* < 0.01) only when using ASVs, while nitrate and chlorophyll *a* were significant when using OTU-97%.

### NBC versus BLCA for taxonomic classification.

**FIG 6:**
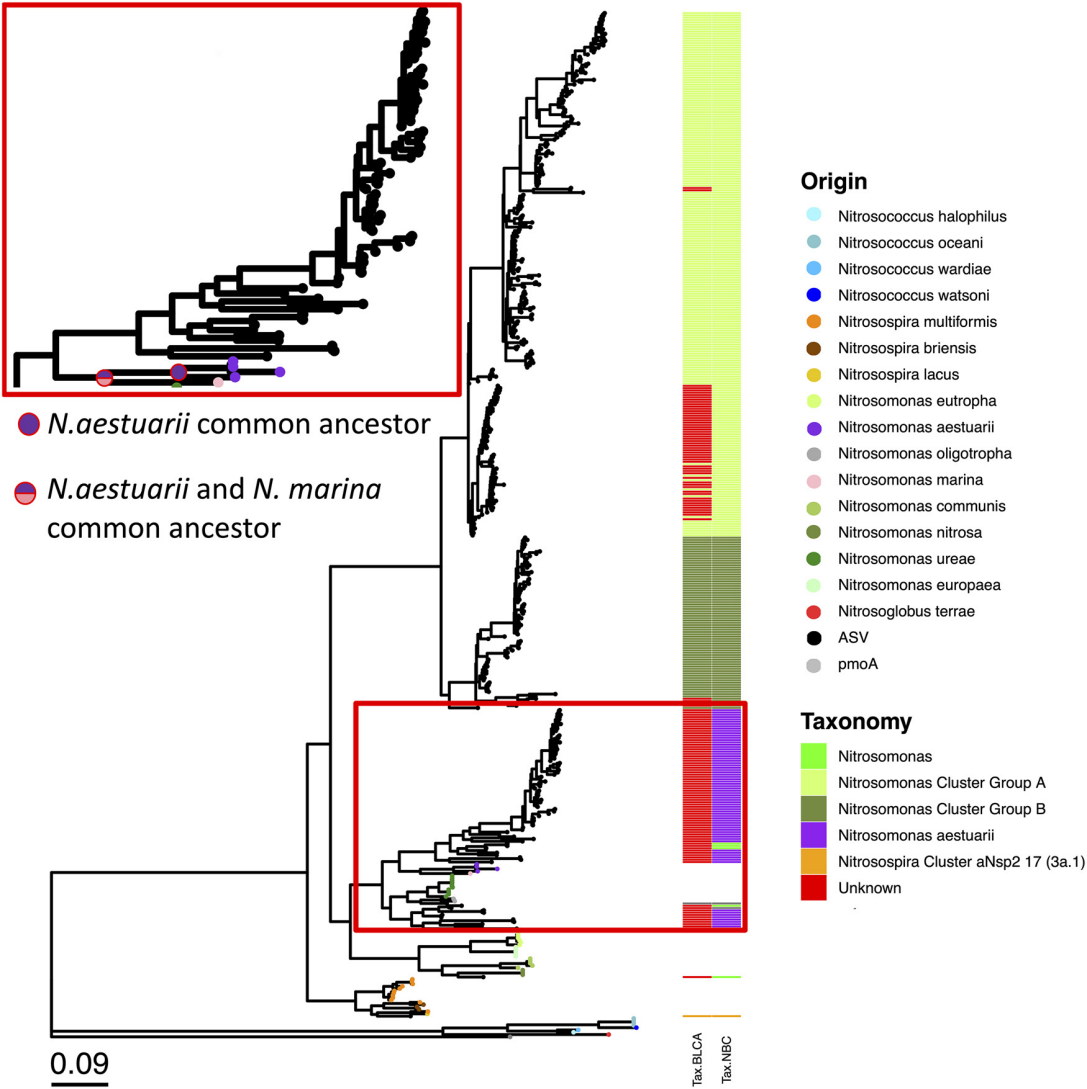
NBC versus BLCA for the taxonomic assignment of AOB *amoA* ASVs. The taxonomy of representative sequences is indicated by the color of the tips on the tree. Taxonomy assigned by NBC (Tax.NBC) and BLCA (Tax.BLCA) for each ASV is represented at the right of the tree. The main point of disagreement between NBC and BLCA is shown by the red rectangle (see the zoomed-in view at the top left).

For the majority of genes studied here, neither the NBC nor the BLCA method performed well, with the majority of ASVs being unassigned at the species level when using the Fungene database. This is likely an indication of the lack of environmental sequences with species-level-defined taxonomy in this database. The only exception was for *nrfA*, with the NBC approach resulting in only 17.27% of ASVs being unassigned, versus the BLCA method, in which 100% were unassigned. Both NBC and BLCA performed better when a custom database was used to assign taxonomy (AOA and AOB *amoA*). In this case, BLCA performed slightly better than NBC for AOA *amoA*, with 72.89% and 90.85% of ASVs being unassigned with BLCA and NBC, respectively. Inversely, for AOB *amoA*, NBC performed slightly better than BLCA, with 0.01% and 6.68% of ASVs being unassigned with NBC and BLCA, respectively (Table 4). To determine what caused these differences for AOB *amoA*, a phylogenetic tree was constructed with AOB *amoA* ASVs and sequences from known AOB isolates downloaded from the NBCI database. The main differences in the taxonomic assignments for AOB *amoA* ASVs were for some sequences assigned as Nitrosomonas aestuarii with NBC that were unassigned using BLCA (Fig. 6).

**TABLE 4:** Performances of the NBC and BLCA methods for taxonomic assignment (ASVs)

Target	Taxonomic method	Database	% unassigned species
AOB *amoA*	NBC	Fungene	100
		Custom	0.01
	BLCA	Fungene	100
		Custom	6.68

AOA *amoA*	NBC	Fungene	84.72
		Custom	90.85
	BLCA	Fungene	100
		Custom	72.89

*nxrB*	NBC	Fungene	78.92
	BLCA		60.02

*nirK*	NBC	Fungene	99.91
	BLCA		99.95

*nirS*	NBC	Fungene	100
	BLCA		100

*nrfA*	NBC	Fungene	17.27
	BLCA		100

## DISCUSSION

The aim of this study was to evaluate the effect of the selection of different OTU similarity thresholds versus the ASV approach for amplicon sequence data processing. This was tested using a suite of functional genes targeting different pathways of the nitrogen cycle. We determined the effect that these choices had on alpha and beta diversity values and subsequent CCA. We also examined the effect of the approach taken for the taxonomic assignment of functional genes using the nitrogen cycle communities from ridges and runnels of the Montportail-Brouage mudflat as a case study.

The choice of amplicon reconstruction method had a significant effect on biological observations (Fig. 2, 4, and 5). As a result, the conclusions of this study based on OTUs (with different similarity thresholds) or ASVs lead to different ecological conclusions. Indeed, when comparing WUniFrac distances using the ASV denoising approach, we conclude that there was a significant effect of the ridge/runnel sedimentary structures on AOA *amoA*, *nirS*, and *nrfA*. On the other hand, we conclude that there was a nonsignificant effect on AOA *amoA* with OTU-95% and OTU-90% and *nirS* with OTU-97% and OTU-95%. For AOB *amoA*, using the ASV method, we conclude that there was a nonsignificant effect of the ridge/runnel structures and a significant effect with OTU-85% (WUniFrac distances) and OTU-97% (UniFrac distances). To further understand the effect of the choice of ASVs versus OTUs on phylogenetic resolution, phylogenetic trees were constructed from ASVs and OTUs. For all target genes, an increase in dissimilarity was observed as the number of ASVs/OTUs compared increased, indicating that the choice of amplicon reconstruction method modifies the phylogeny of representative sequences (see Fig. S2 in the supplemental material).

The results from this study show the strong effect that the processing method has on the interpretation and biological understanding of sequencing data. This further illustrates the need for a standardized protocol for amplicon data processing to facilitate comparisons of data between studies. To date, there is no consensus as to which threshold to use to construct OTUs for the same gene amplified with the same primers (Table 1), and we therefore argue that ASVs, which do not require user-defined similarity thresholds, offer a better chance to achieve such standardization. The ASV method also generally resulted in a higher percentage of raw reads retained than with the OTU method, especially for AOA *amoA* and *nrfA*. Previous studies have shown that retaining more reads after amplicon processing improves the accuracy of microbiome analyses, especially for low-abundance species (43). This further strengthens our recommendation to use ASVs for functional gene HTS data processing. We further showed that the choice of amplicon reconstruction method affects the outcomes of multivariate analyses, which are routinely used to inform associations between biological assemblages and environmental parameters. For example, research on nitrification in the environment often seeks to determine the extent to which ammonia, pH, salinity, and temperature, etc., are significant drivers of the niche differentiation between AOA and AOB (7–9, 44–51). This study clearly shows that the influence of environmental parameters on ammonia-oxidizing communities is dependent on the amplicon reconstruction method used. A consensus standardized method needs to be adopted in molecular microbial ecology to allow metareviews of current literature and the identification of ecological patterns that are not study-dependent (51, 52).

Other studies have reported that for *16S rRNA* gene amplicon sequencing, ecological patterns are robust to the choice of OTUs versus ASVs (32, 35, 52, 53). It could therefore be hypothesized that the difference between the *16S rRNA* gene as reported elsewhere and functional genes is due to a resolution effect: when targeting a high-diversity gene such as the *16S rRNA* gene by amplicon sequencing, we obtain an overall picture of the microbial community. Therefore, the use of OTUs (low resolution) or ASVs (high resolution) matters less because we still see the overall trends in the bacterial community. However, when investigating a phylogenetically tight group, we are already zoomed in on a small part of that microbial community, which explains why we need a higher resolution, i.e., ASVs, to differentiate each member of the community.

This study also provides a comparison between NBC and BLCA for the taxonomic assignment of functional gene sequences. We found that the BLCA method performed better than the NBC for AOA *amoA* when using a custom database, and the inverse was found for AOB *amoA* due to some sequences being assigned to Nitrosomonas aestuarii when using NBC and being unassigned when using BLCA. When comparing these sequences to known representatives, it was observed that known AOB *amoA* sequences from Nitrosomonas aestuarii were clustered, and the common ancestor for all N. aestuarii strains did not include any of these ASVs. In fact, based on this tree, *N. aestuarii* and Nitrosomonas marina share an ancestor that does not include any ASV sequences. Based on this phylogeny, these ASVs could therefore not be members of the *N. aestuarii* group (Fig. 6). We conclude that for AOB *amoA*, the BLCA method, despite resulting in a lower number of sequences identified to the species level, resulted in a classification that made more sense from a phylogenetic point of view. This result is coherent with previous research showing the superiority of BLCA versus NBC for the taxonomic assignment of *16S rRNA* gene reads (40). However, the BLCA method performed much worse than NBC for the taxonomic assignment of *nrfA* sequences, with the vast majority (98.8%) being unassigned sequences, versus 18.8% with NBC. This likely reflects limitations in the database used rather than a problem with the BLCA method itself. Indeed, the majority of *nrfA* sequences in the Fungene database are full-length sequences originating from cultured microorganisms, which likely differ from sequences retrieved from the environment. To test this hypothesis, a phylogenetic tree was drawn using the top 200 *nrfA* ASVs found in this study and sequences from the reference database (covering 220 different genera). ASV sequences and representatives from the reference database generally formed separate clusters in the tree (Fig. S3). As a result, it can be expected that when ASVs are subjected to a BLAST search against the reference database, the significant matches do not share an ancestor at the genus level, and as a result, the BLCA algorithm cannot assign taxonomy at the genus or species level. In summary, the results from this study indicate that when the reference database is relevant to the sequences amplified (e.g., reference AOB *amoA* sequences from marine sediments to assign AOB *amoA* ASVs from marine sediments), the BLCA method is the best approach. On the other hand, if the reference database consists of sequences more dissimilar to the one retrieved from the environment, the NBC method might be more advisable to obtain taxonomic information, but the accuracy of this taxonomy might be low. ASVs have substantial merits for the analysis of functional genes for which OTU similarity values are unknown, and in general, the field is shifting to ASV-based resolution of amplicons (e.g., see reference 13). Yet the reference databases used to taxonomically place the amplicons are still based on traditional threshold-based clustering and should improve as more and more ASV-based studies become available in the future.

10.1128/msphere.00324-22.4 FIG S3 Phylogenetic relationship between the top 200 most abundant *nrfA* ASVs and sequences from the reference database covering 220 different genera. Download FIG S3, PDF file, 0.05 MB. Copyright © 2022 Cholet et al. 2022 Cholet et al. https://creativecommons.org/licenses/by/4.0/ This content is distributed under the terms of the Creative Commons Attribution 4.0 International license.

Currently, there is a shift in microbial ecology from OTUs to ASVs, and this approach has now been widely adopted for *16S rRNA* gene studies (33, 36, 54). The use of ASVs frees us from using similarity thresholds and produces relevant sequences that can be directly compared between studies; we suggest that it offers a pragmatic approach for the standardization of functional gene amplicon sequencing data sets. However, Schloss (53) recently showed that the use of ASVs for the *16S rRNA* gene can artificially split bacterial genomes as copies of the gene in a single genome typically do not share 100% similarity. Several functional genes are also present in more than one copy in a genome, and their sequences can be slightly different (55, 56). In this case, a similar split will take place, and genes from the same organism will be separated. Merging functional genes based on their amino acid (AA) sequence similarity could be an option to reduce the risk of such artificial splits while conserving the functional diversity within the data set. On the other hand, merging at the protein level might overshadow differences between species/strains where synonymous mutations have accumulated. Furthermore, the chance of generating identical protein sequences will increase as the length of the amplified region decreases. For short amplicons with low diversity, merging at the protein level might therefore result in several species being merged. Further work is needed to understand how functional diversity informs ecological processes.

### Conclusion.

Besides the obvious advantage of not relying on an arbitrary threshold, the ASV method reflects the sequence diversity of a given functional gene in the environment. This, alongside the ability to compare ASVs among different studies, makes ASVs more practical than OTUs for functional gene analysis in environmental microbiology. Finally, we recommend the use of a relevant database that closely resembles the expected sequences from the environment studied along with the BLCA method for taxonomic classification. If such a database cannot be obtained, the NBC approach might yield better results.

## MATERIALS AND METHODS

### Similarity thresholds from the literature used to cluster OTUs for nitrogen cycle genes.

Similarity thresholds were selected based on a literature review for all amplicon-based nitrogen cycle studies. The list provided in Table 1 is, to the best of our knowledge, an exhaustive representation of OTU similarity thresholds used for the genes tested.

### Sample collection, physicochemical measurements, PCR, and Illumina sequencing.

**FIG 7:**
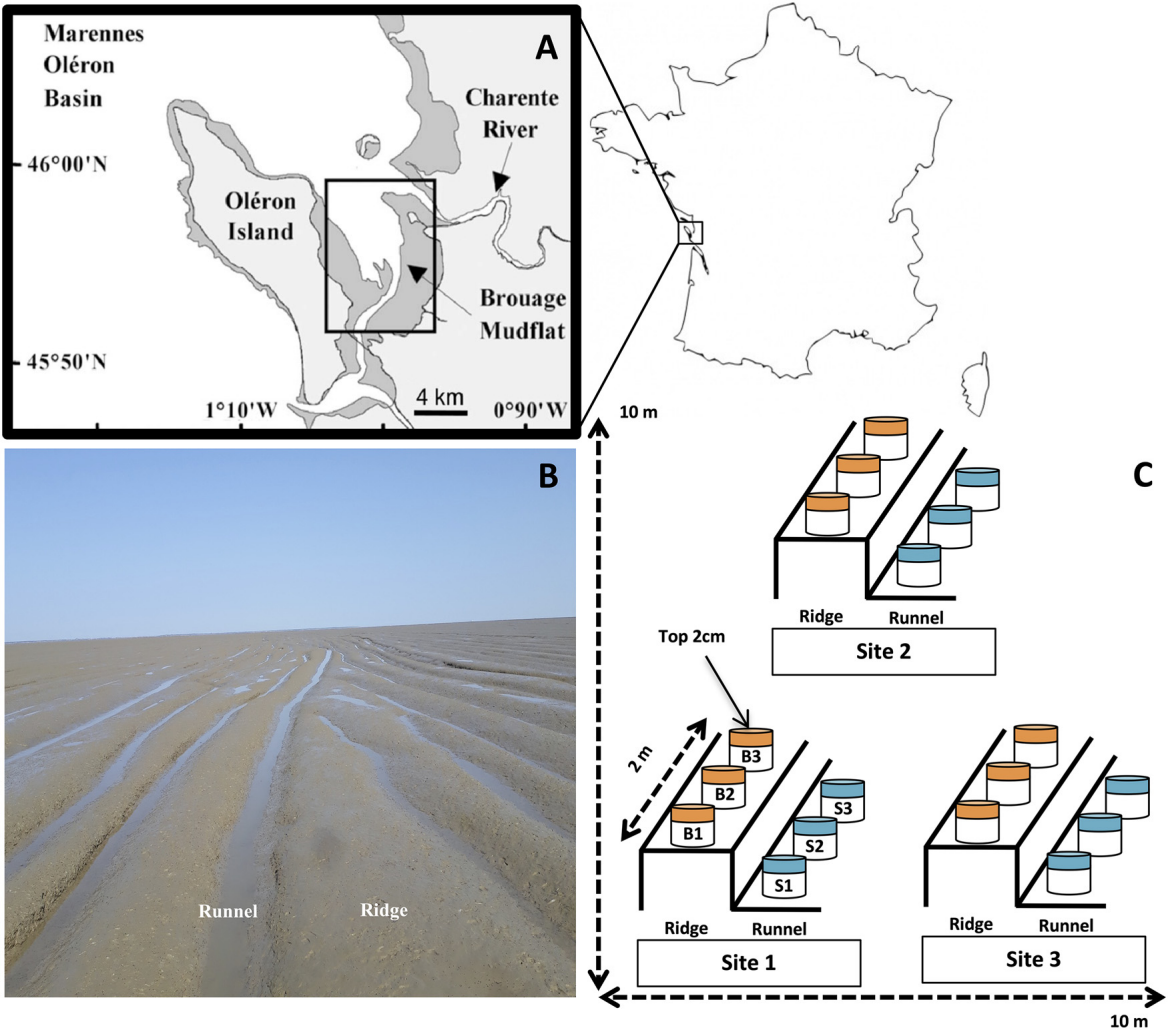
Sediment sampling of ridges and runnels on the Brouage mudflat. (A) Map showing the location of the Brouage mudflat on the Marennes-Oléron Bay on the French Atlantic coast. (B) Parallel ridge-runnel sedimentary structures that characterize the intertidal mudflat. (C) Schematic of the sampling plan. Three ridges-runnels within ~25 m^2^ were sampled. For each ridge-runnel structure, replicate (*n* = 3) sediment cores (2-cm depth) were taken from the ridges and runnels along an ~2-m transect.

Triplicate surface mud cores (0 to 2 cm) were sampled in July 2016 from the Montportail-Brouage mudflat, France, from three different ridges and runnels within 27.41 m^2^ (location 1 [L1], 45 54 31.50 N, 001 05 14.60 W; location 2 [L2], 45 54 31.70 N, 001 0 514.20 W; location 3 [L3], 45 54 31.50 N, 001 05 14.20 W) (57, 58) at the VASIREMI station (Fig. 7). Sediment was homogenized, collected in sterile 5-mL syringes, flash-frozen, and stored at −80°C until subsequent use. Biophysicochemical parameters were measured as described previously (58); detailed procedures are provided in Text S1 in the supplemental material. DNA extraction was carried out using a modification of a protocol developed previously (59), and PCR of the *amoA* (bacteria and archaea), nxrB, *nirSK*, and *nrfA* genes was carried out as detailed in Table 5. Illumina amplicon sequencing library preparation was carried out as described previously by Cholet et al. (10), using the Nextera XT index kit (Illumina, UK). Products were pooled at equimolar concentrations and submitted to the Earlham Institute (Norwich, UK) for Illumina MiSeq sequencing (300PE (Paired-End); 22 million reads/lane). Detailed protocols are provided in Text S1.

**TABLE 5:** List of primers and corresponding PCR conditions used in this study

Primer	Sequence (5′–3′)	Orientation	Target	Length (bp)	PCR conditions	Reference
BacamoA-1F	GGGGHTTYTACTGGTGGT	Forward	*amoA*; ammonia oxidation (bacteria)	491	95°C for 15 min; 32 cycles of 94°C for 30 s, 47°C for 40 s, and 72°C for 1 min; and 72°C for 10 min	94
BacamoA-2R	CCCCTCBGSAAAVCCTTCTTC	Reverse				

Arch-amoWAF	CTGAYTGGGCYTGGACATC	Forward	*amoA*; ammonia oxidation (archaea)	256	95°C for 15 min; 35 cycles of 95°C for 30 s, 58°C for 40 s, and 72°C for 1 min; and 72°C for 10 min	95
Arch-amoWAR	TTCTTCTTTGTTGCCCAGTA	Reverse				

nirK FlaCu	ATCATGGTSCTGCCGCG	Forward	*nirK*; nitrite reductase	472	95°C for 15 min; 30 cycles of 95°C for 30 s, 57°C for 30 s, and 72°C for 40 s; and 72°C for 10 min	96
nirK R3Cu	GCCTCGATCAGRTTGTGGTT	Reverse				
nirS1F	CCTAYTGGCCGGCRCART	Forward	*nirS*; nitrite reductase	256		97
nirS 3R	GCCGCCGTCRTGVAGGAA	Reverse				

nrfAF2aw	CARTGYCAYGTBGARTA	Forward	*nrfA*; nitrite reduction (DNRA)	250	95°C for 15 min; 35 cycles of 94°C for 30 s, 50°C for 20 s, and 72°C for 40 s; and 72°C for 10 min	98
nrfAR1	TWNGGCATRTGRCARTC	Reverse				

nxrB169f	TACATGTGGTGGAACA	Forward	*nxrB*; nitrite oxidation (*Nitrospira*)	485	95°C for 15 min; 30 cycles of 94°C for 30 s, 56°C for 30 s, and 72°C for 1 min; and 72°C for 10 min	99
nxrB638r	CGGTTCTGGTCRATCA	Reverse				

10.1128/msphere.00324-22.1 TEXT S1 Supplemental methods. Download Text S1, DOCX file, 0.04 MB. Copyright © 2022 Cholet et al. 2022 Cholet et al. https://creativecommons.org/licenses/by/4.0/ This content is distributed under the terms of the Creative Commons Attribution 4.0 International license.

### OTU and ASV construction.

To construct OTUs, paired-end reads were trimmed and filtered with Sickle v1.2 (60) using a sliding window and trimming regions where the average base quality was below 20. A 10-bp threshold was used to discard reads below this length. BayesHammer (60) and Spades v2.5.0 assembler were used to error correct paired-end reads, followed by pandaseq v2.4 with a minimum overlap of 20 bp to assemble the forward and reverse reads into a single sequence. The choice of software was a result of our recent work (61, 62) where it was shown that the above-described strategy of read trimming followed by error correction and overlapping reads reduces the substitution rates significantly. After having obtained the consensus sequences from each sample, the VSEARCH (v2.3.4) pipeline (all of these steps are documented at https://github.com/torognes/vsearch/wiki/VSEARCH-pipeline) was used for OTU construction. Reads were pooled from different samples, and barcodes were added to keep an account of the samples that the reads originated from. Reads were then dereplicated and sorted by decreasing abundance, and singletons were discarded. In the next step, the reads were clustered based on different similarity thresholds (97%, 95%, 90%, and 85% for AOA and AOB *amoA*; 97%, 95%, 90%, and 83% or 82% for *nirK* [83%] and *nirS* [82%]; and 97% for *nxrB* and *nrfA*), followed by the removal of clusters that have chimeric models built from more abundant reads (–uchime_denovo option in VSEARCH). A few chimeras may be missed, especially if they have parents that are absent from the reads or are present at a very low abundance. Therefore, in the next step, we used a reference-based chimera-filtering step (–uchime_ref option in VSEARCH) using the reference databases created as described above. The quality-filtered barcoded reads were matched against clean OTUs with different similarity thresholds to generate OTU tables.

Amplicon sequence variants (ASVs) were constructed in R using the DADA2 package, according to the tutorial at https://benjjneb.github.io/dada2/tutorial.html. First, quality trimming was done using filterAndTrim(). The trimRight and trimLeft parameters, used to trim the 3′ ends of reads and primer sequences, respectively, were adjusted for each target gene, as listed in Table S1. The 3′-end trimming length was adjusted to remove low-quality portions of the reads while still allowing a minimum of a 20-bp overlap between the forward and reverse reads, except for AOB *amoA* and *nxrB*, where the minimum overlap was reduced to 4 bp. Error models were generated against the filtered forward and reverse reads using the learnErrors() function. Reads were then dereplicated using the derepFastq() function, and ASVs were inferred using the dada() function. Forward and reverse reads were merged using mergePairs(). A sequence table was generated using the makeSequenceTable() function, and chimeras were removed using the removeBimeraDenovo() function. A count table was then generated, and distances between the representative ASVs were inferred by aligning the sequences using Mafft (63) and constructing a phylogenetic tree using FastTree (64). The R script used for ASV construction is available in Text S1.

10.1128/msphere.00324-22.5 TABLE S1 Parameters for ASV quality filtering. Download Table S1, DOCX file, 0.01 MB. Copyright © 2022 Cholet et al. 2022 Cholet et al. https://creativecommons.org/licenses/by/4.0/ This content is distributed under the terms of the Creative Commons Attribution 4.0 International license.

### Taxonomic assignment by NBC and BLCA.

For each nitrogen cycle gene, reference sequences (nucleotides) were downloaded from Fungene (http://fungene.cme.msu.edu/); for AOA and AOB *amoA*, a second database was constructed by downloading the sequences corresponding to the different clusters defined previously by Zhang et al. (8). Subsequently, the R rentrez package (65) was used to obtain taxonomic information at different levels, generating a taxonomy file. The FASTA file and the corresponding taxonomy file were formatted to work with Qiime (66).

To assign taxonomy to the representative ASVs, two different approaches were used: representative ASVs were classified using a naive Bayesian classifier (NBC) k-mer classifier (Qiime feature classifier classify-sklearn) or the Bayesian lowest common ancestor (BLCA) (39) against the reference databases, using default parameters. A detailed protocol for BLCA can be found at https://github.com/qunfengdong/BLCA. Count tables, generated in the previous step, and taxonomy tables were combined to generate biom files using Qiime (66) (https://qiime2.org) (biom add-metadata), and the phyloseq package was used to load these biom files in R.

### Amplicon read quality check.

After OTU and ASV construction, a quality check step was undertaken to ensure the reliability of the data. First, the correct reading frame was determined for OTU/ASV nucleotide sequences (from which primer sequences were removed) using the EXPASY translate online tool (https://web.expasy.org/translate/) and performing a BLAST search for the six different proteins against the standard nonredundant (nr) database using the BLASTp algorithm with default settings. All nucleotide sequences were then translated to protein sequences using the translate() function from the seqinr R package (67). The resulting amino acid (AA) sequences were separated into two categories: one containing AA sequences of the expected length (Table S2) and one containing AA sequences longer or shorter than the expected length. The latter sequences were submitted to a BLAST search against the standard nr database using the BLASTp algorithm. Sequences that matched the expected enzyme were then reintegrated into the first category of verified sequences. For downstream statistical analyses in R, abundance tables and phylogenetic trees were curated by retaining only these verified sequences.

10.1128/msphere.00324-22.6 TABLE S2 Expected length of the target genes. The expected lengths of both the nucleotide and amino acid (AA) sequences are calculated for the amplicon without primer sequences. Download Table S2, DOCX file, 0.01 MB. Copyright © 2022 Cholet et al. 2022 Cholet et al. https://creativecommons.org/licenses/by/4.0/ This content is distributed under the terms of the Creative Commons Attribution 4.0 International license.

### Downstream statistical analyses.

**(i) Alpha diversity indices.** The vegan package (68) was used to calculate richness (vegan::rarefy), Shannon entropy, and Simpson diversity (vegan::diversity) separately in ridges and runnels after rarefaction to 10,000 reads. Visualization was achieved using the ggplot2 package (https://cran.r-project.org/web/packages/ggplot2/index.html).

**(ii) Rarefaction curves.** Average abundance tables were generated by calculating the average abundance of each OTU/ASV in ridges and runnels. Rarefaction curves were then computed using the iNEXT package (69). Visualization was achieved using ggiNEXT().

**(iii) Beta diversity indices.** Abundance tables and phylogenetic trees were combined using phyloseq’s merge_phyloseq() function. Distances (Bray-Curtis/UniFrac/WUniFrac) between samples were calculated using phyloseq’s phyloseq::distance() function and used for PERMANOVAs using vegan’s adonis() function to determine if the sediment type (ridges and runnels) had a significant effect on the community composition. Visualization was achieved using the ggplot2 package (https://cran.r-project.org/web/packages/ggplot2/index.html).

**(iv) Mantel correlation tests.** Distances (Bray-Curtis/UniFrac/WUniFrac) between samples were calculated using phyloseq’s phyloseq::distance() function, and the correlations between distance matrices were calculated using mantel.rtest() (nrepet = 9,999, “two-sided”) from the ade4 package in R (https://www.jstatsoft.org/article/view/v086i01).

**(v) Canonical correspondence analysis.** To find significant drivers of the nitrogen-cycling communities, a canonical correspondence analysis (CCA) was carried out in R. First, the abundance tables (i.e., the OTU/ASV counts for each target in each sample) were normalized using the Hellinger transformation (70). Next, the parameter table (i.e., the table containing the biophysicochemical parameters for each sample) was normalized by centering and reduction. The CCA was then computed using the cca function from the R vegan package (68), with the standardized parameter table as the explanatory table and the Hellinger-transformed OTU/ASV abundance table as the response table. Variable selection was then carried out using the ordistep function (vegan package) with the option direction=“both,” allowing simultaneous backward and forward selection to find significant drivers for each target gene.

**(vi) Comparison of phylogenetic trees.** To evaluate the effects of HTS data processing methods on the phylogeny of representative sequences, representative sequences were aligned with closely related reference sequences obtained from the NBCI and/or Fungene database (for a list of reference sequences, see Table S3) using Mafft (63), and phylogenetic trees were generated using FastTree (64). In the first iteration, only the reference sequences and the most abundant ASVs/OTUs were included in the tree, and distances between trees were calculated using the Robinson-Foulds metric in R using the RF.dist() function from the Phangorn package (71). Additional ASVs/OTUs were added one by one in descending order of abundance. After each addition, the distances between trees were computed. Because the RF.dist() function requires equal numbers of tips in the trees compared, the total number of iterations corresponds to the number of sequences in the abundance table with the lowest number of sequences (e.g., for AOB *amoA*, there were 384 ASVs and 8 OTUs-85%; therefore, a maximum of 8 iterations were done when comparing AOB *amoA* ASV and OTU-85% trees).

10.1128/msphere.00324-22.7 TABLE S3 List of reference sequences for phylogenetic trees. Download Table S3, DOCX file, 0.02 MB. Copyright © 2022 Cholet et al. 2022 Cholet et al. https://creativecommons.org/licenses/by/4.0/ This content is distributed under the terms of the Creative Commons Attribution 4.0 International license.

### Data availability.

Raw reads were submitted to the NCBI database under accession number PRJNA841793.

10.1128/msphere.00324-22.8 TABLE S4 Effect of the amplicon reconstruction method on alpha diversity measures. *Post hoc* tests were carried out using the TukeyHSD() function in R. Download Table S4, DOCX file, 0.02 MB. Copyright © 2022 Cholet et al. 2022 Cholet et al. https://creativecommons.org/licenses/by/4.0/ This content is distributed under the terms of the Creative Commons Attribution 4.0 International license.
