# Supplementary figures and images for: Ecological Observations Based on Functional Gene Sequencing Are Sensitive to the Amplicon Processing Method
Source: mSphere. 2022 Aug 8;7(4):e00324-22. doi: 10.1128/msphere.00324-22 (PMC9429940; doi:10.1128/msphere.00324-22)

1.A. AOA *amoA*

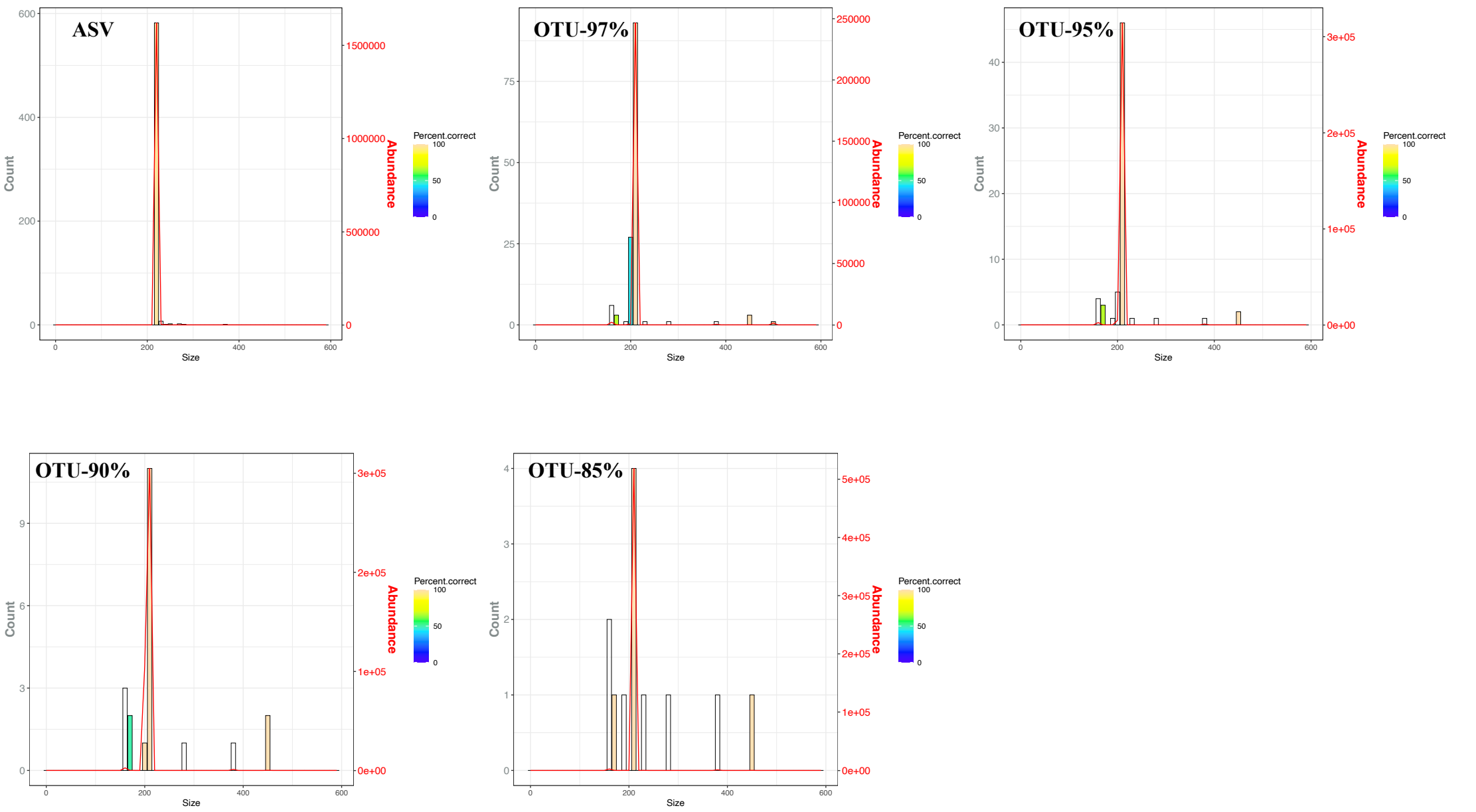

1.B. AOB *amoA*

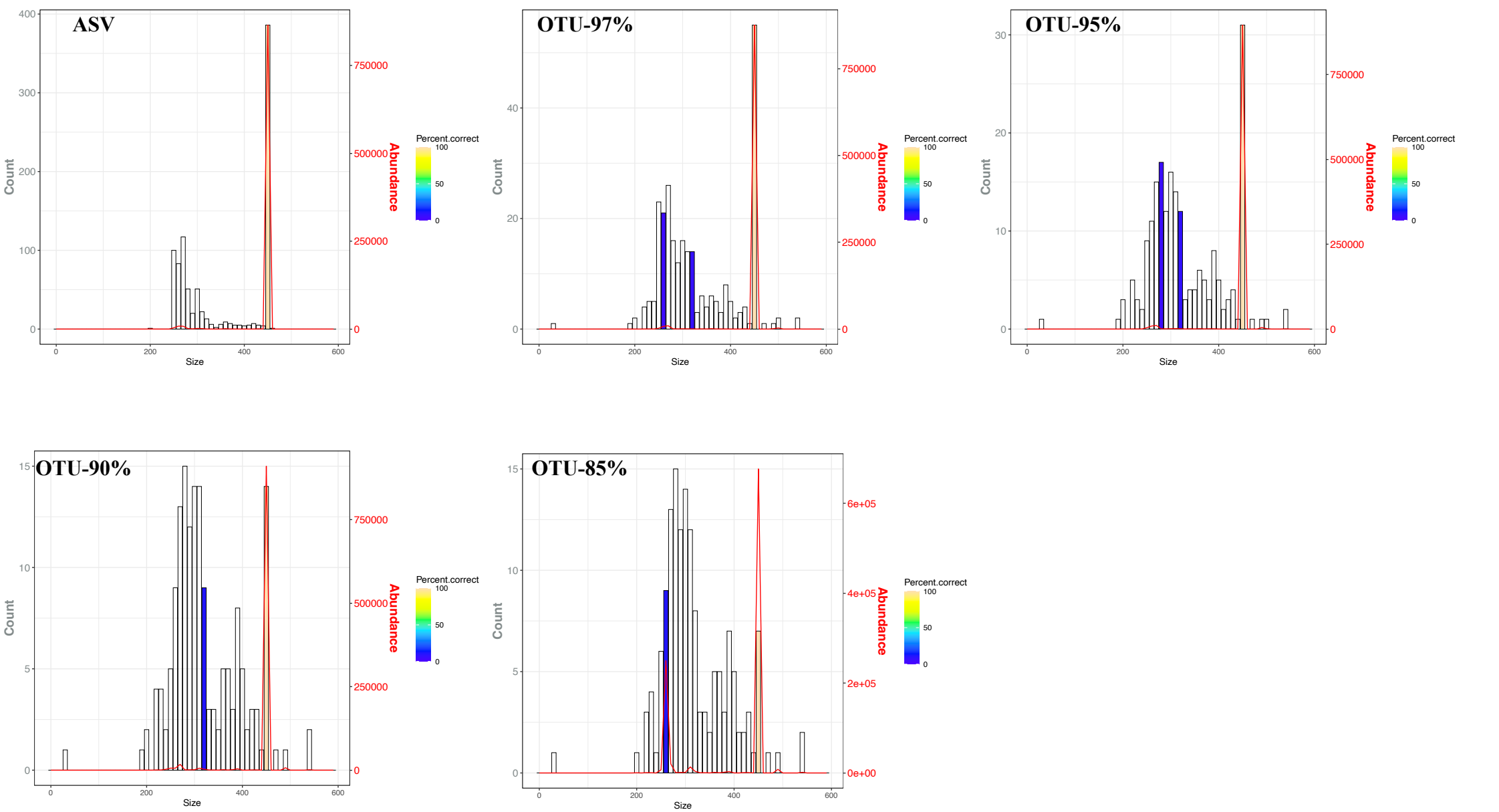

1.C. *nirK*

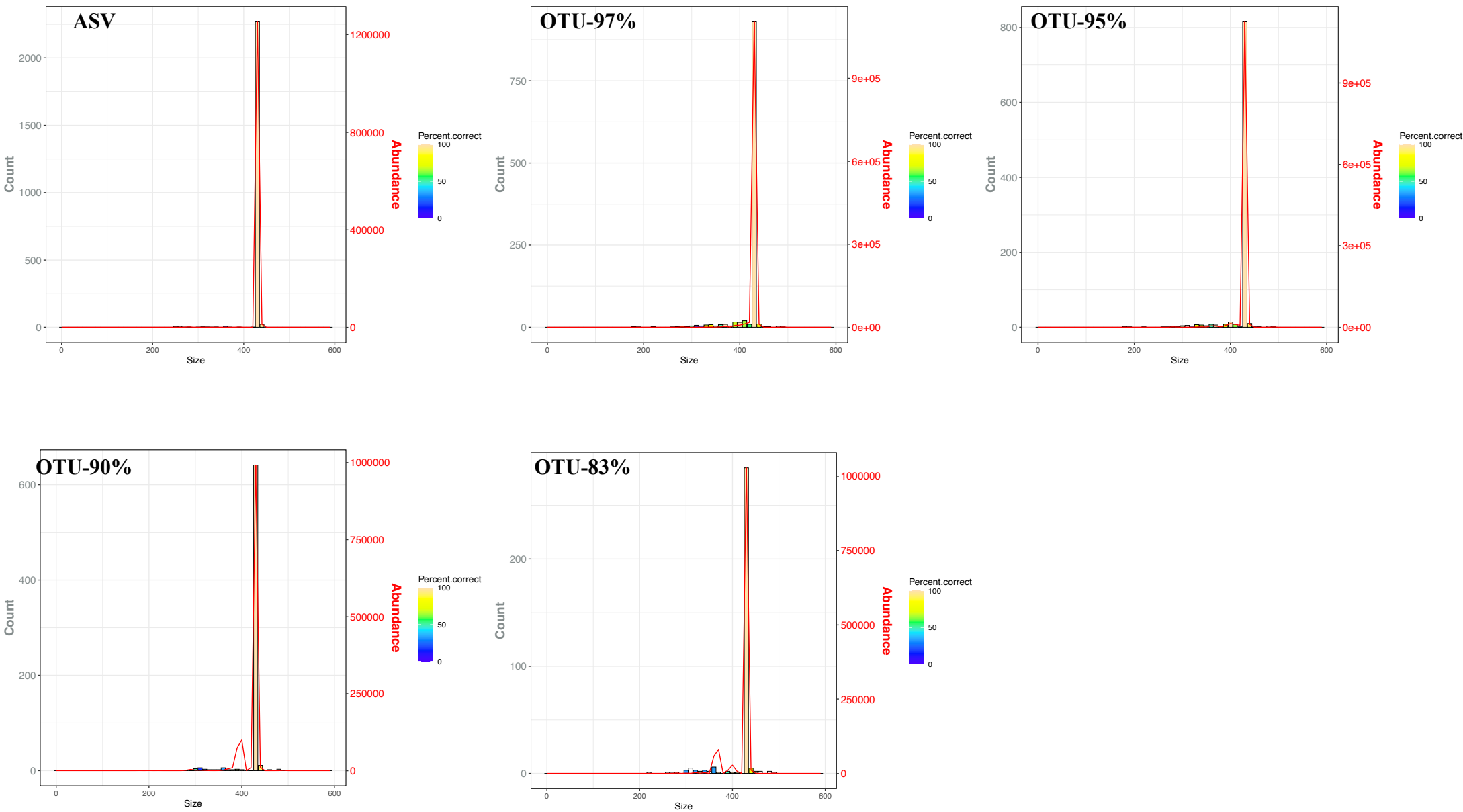

1.D. *nirS*

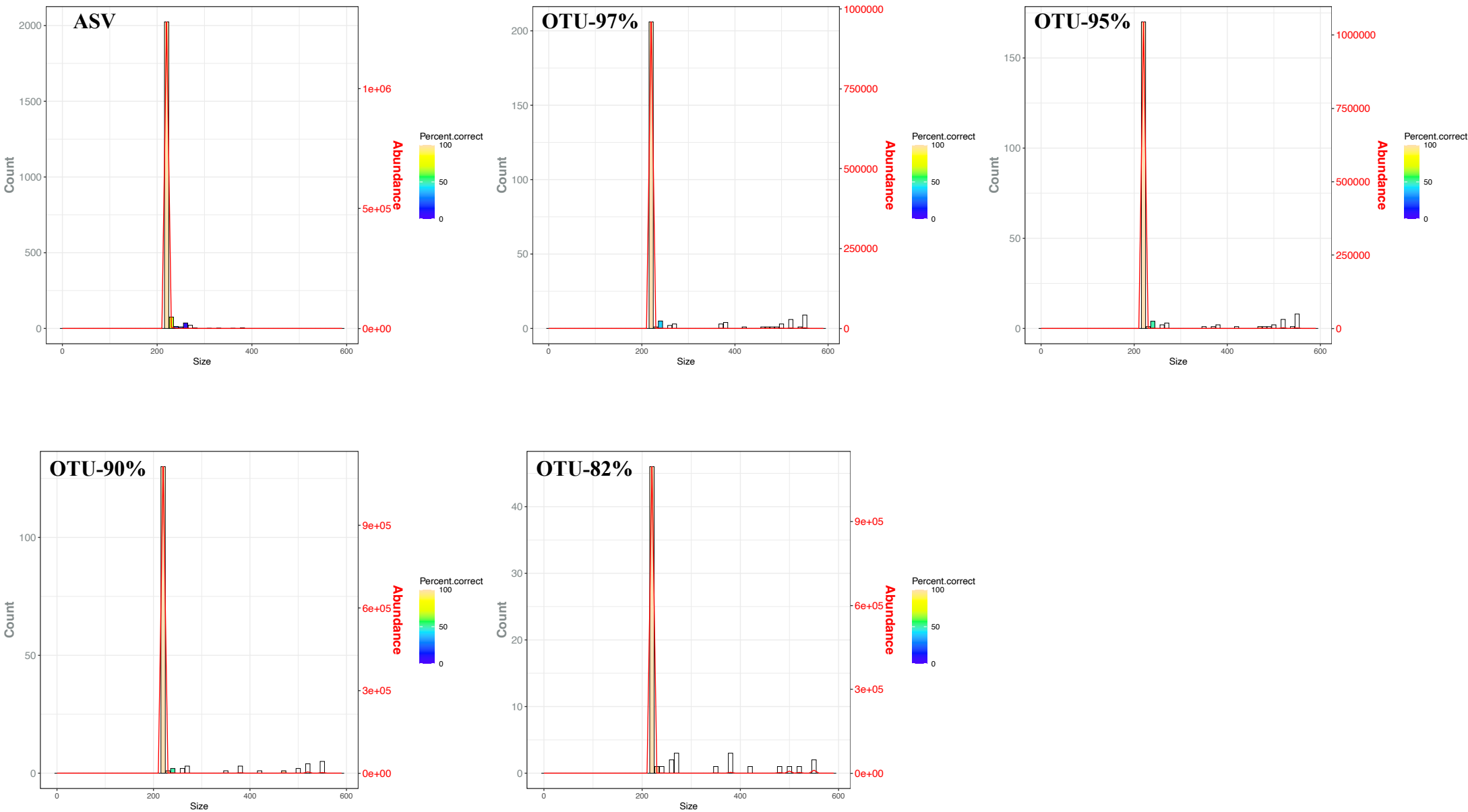

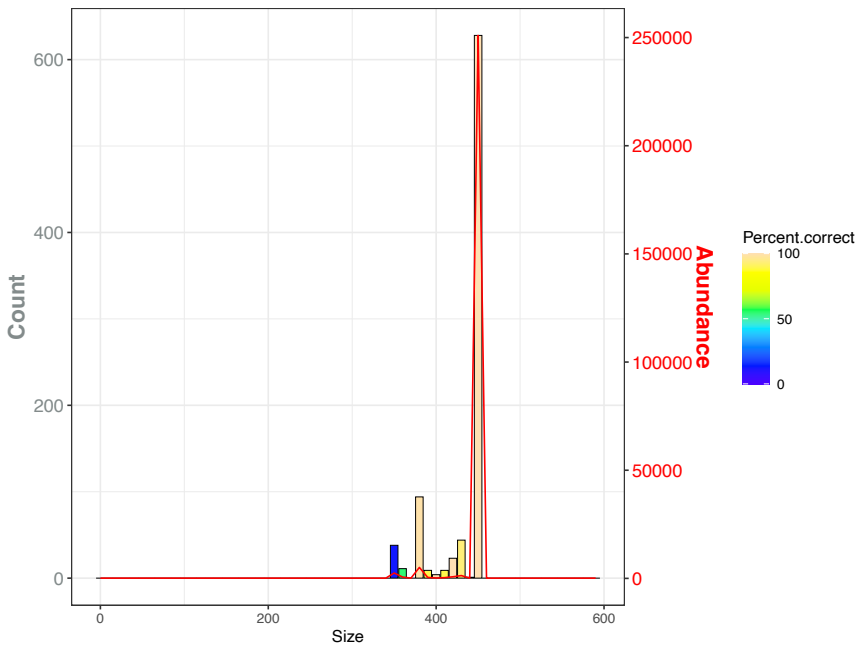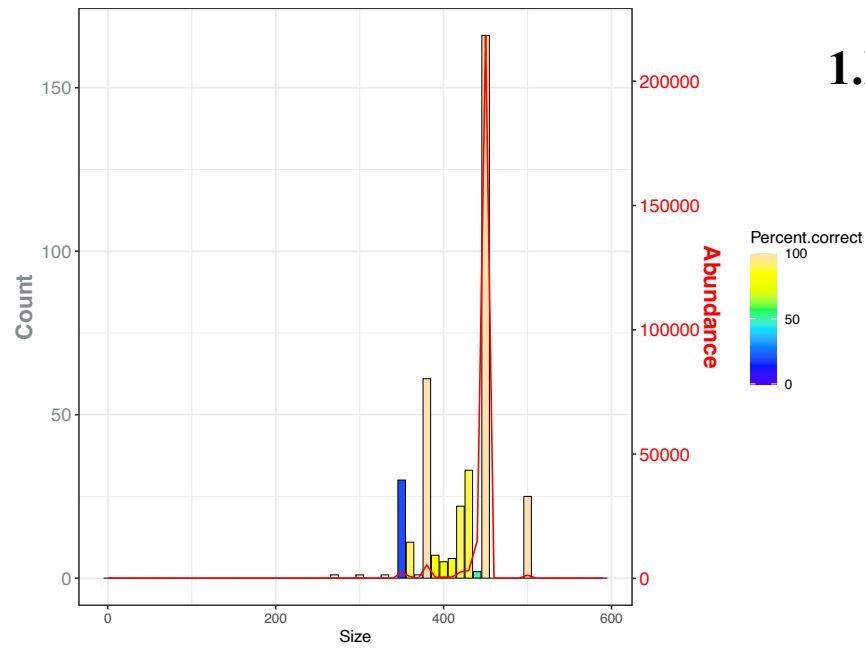

1.E. *nxrB*

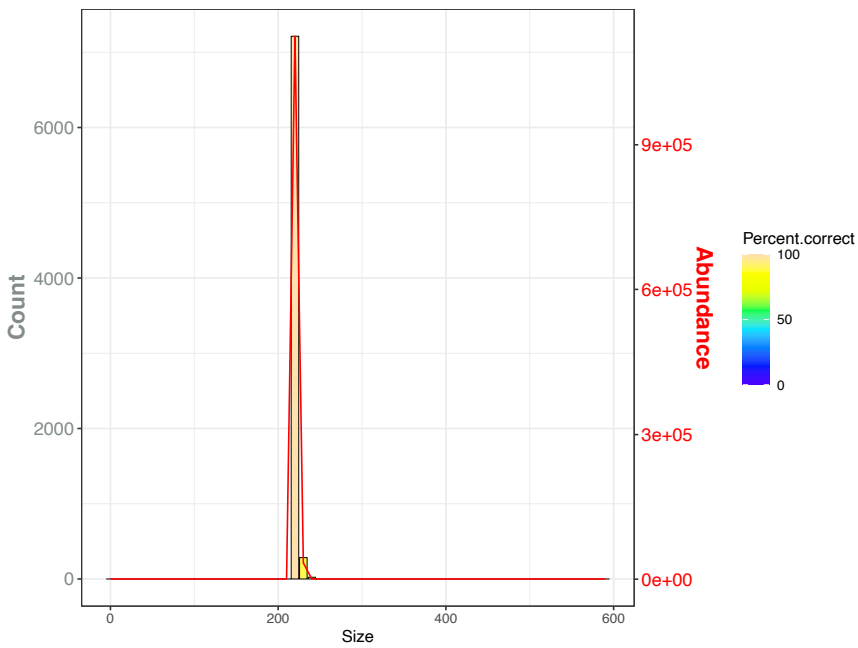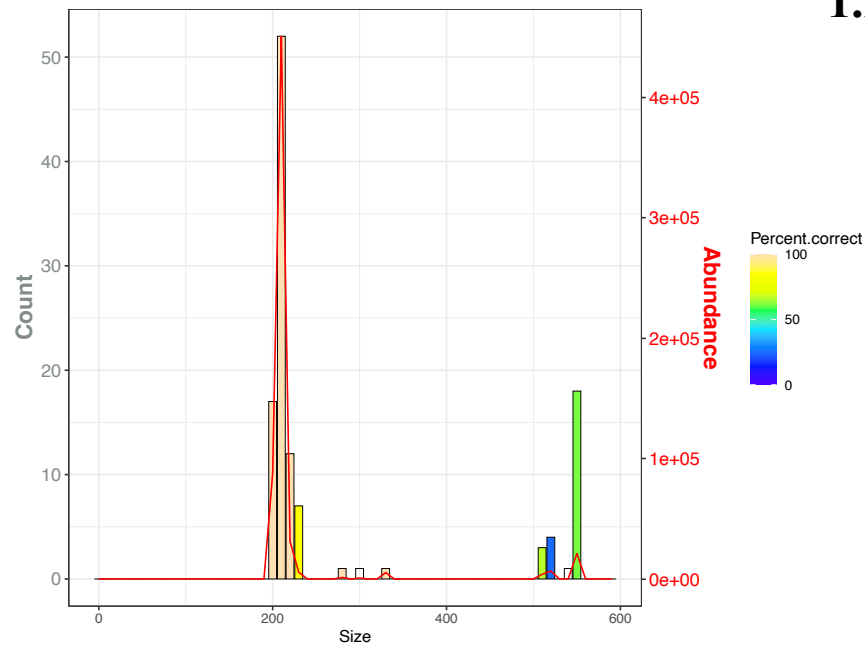

1.F. *nrfA*

Supplement: FIG S1 [file msphere.00324-22-s0001.pdf]

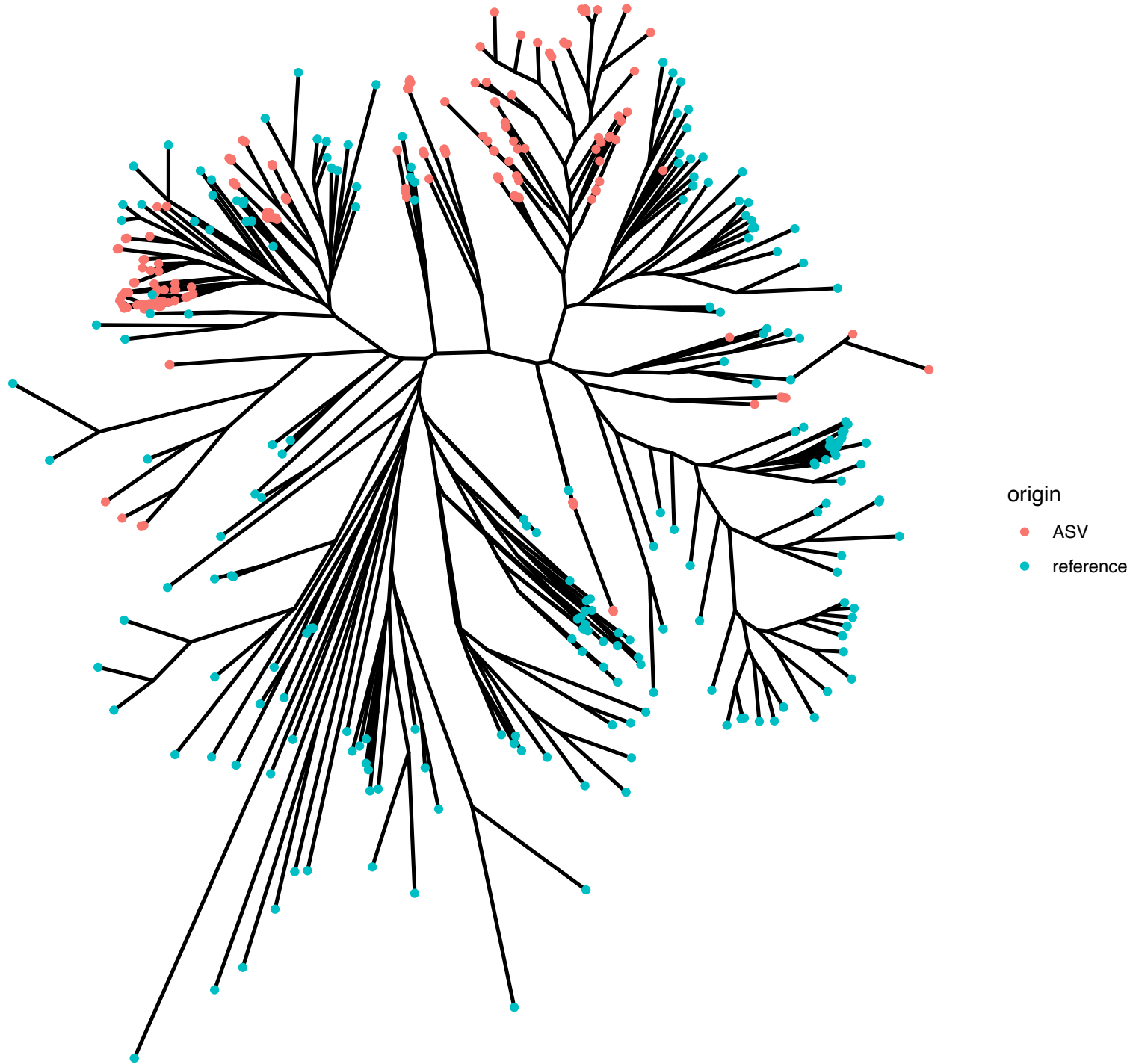

Supplement: FIG S3 [file msphere.00324-22-s0003.pdf]
