# Supplementary material for: Ecological Observations Based on Functional Gene Sequencing Are Sensitive to the Amplicon Processing Method
Source: mSphere. 2022 Aug 8;7(4):e00324-22. doi: 10.1128/msphere.00324-22 (PMC9429940; doi:10.1128/msphere.00324-22)
Supplement: FIG S2 [file msphere.00324-22-s0002.pdf]

2.A. AOA *amoA*

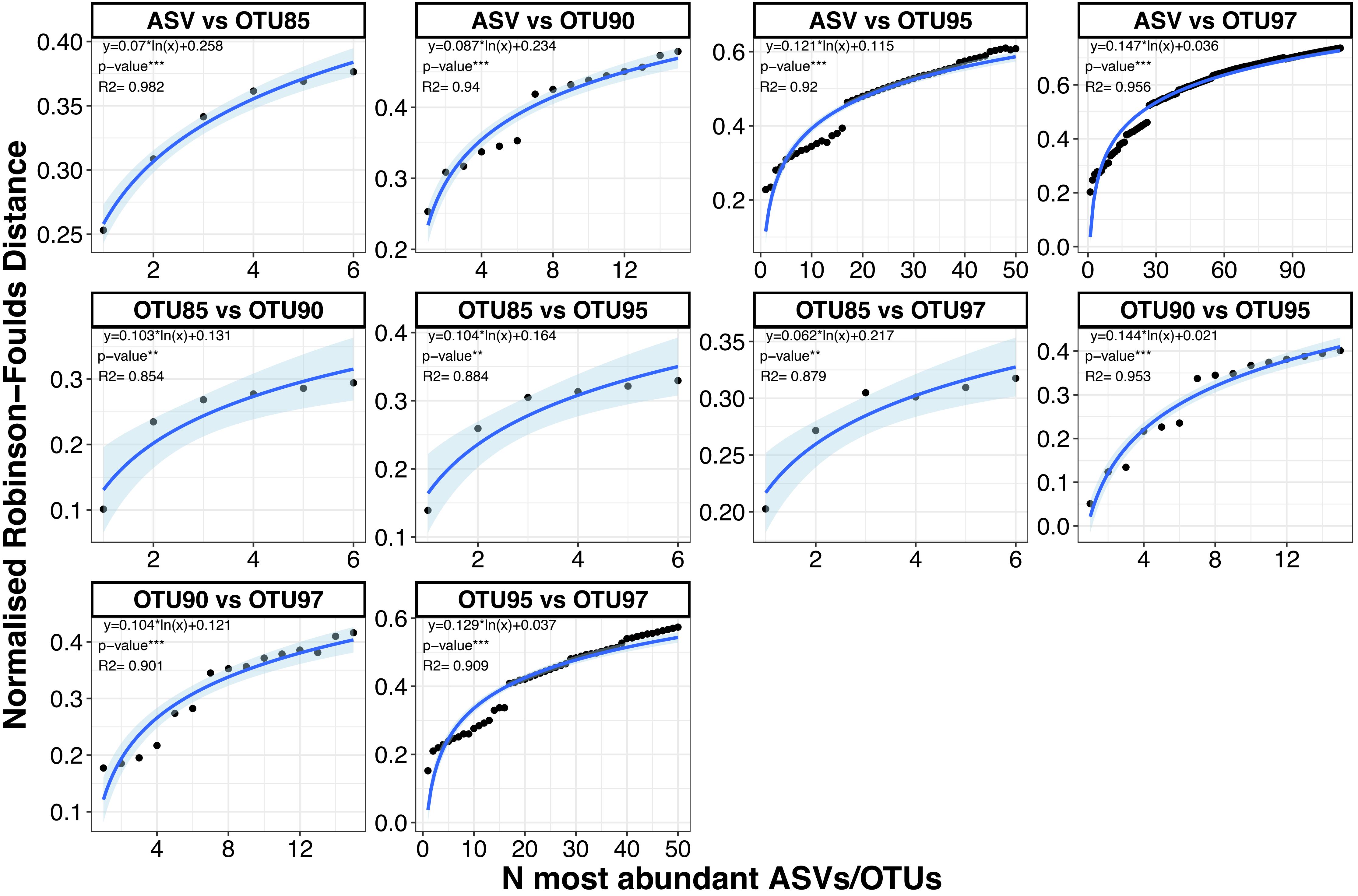
ASV vs OTU85
y=0.07*ln(x)+0.258
p−value***
R2= 0.982
ASV vs OTU90
y=0.087*ln(x)+0.234
p−value***
R2= 0.94
ASV vs OTU95
y=0.121*ln(x)+0.115
p−value***
R2= 0.92
ASV vs OTU97
y=0.147*ln(x)+0.036
p−value***
R2= 0.956
OTU85 vs OTU90
y=0.103*ln(x)+0.131
p−value**
R2= 0.854
OTU85 vs OTU95
y=0.104*ln(x)+0.164
p−value**
R2= 0.884
OTU85 vs OTU97
y=0.062*ln(x)+0.217
p−value**
R2= 0.879
OTU90 vs OTU95
y=0.144*ln(x)+0.021
p−value***
R2= 0.953
OTU90 vs OTU97
y=0.104*ln(x)+0.121
p−value***
R2= 0.901
OTU95 vs OTU97
y=0.129*ln(x)+0.037
p−value***
R2= 0.909
Normalised Robinson−Foulds Distance
N most abundant ASVs/OTUs

2.B. AOB *amoA*

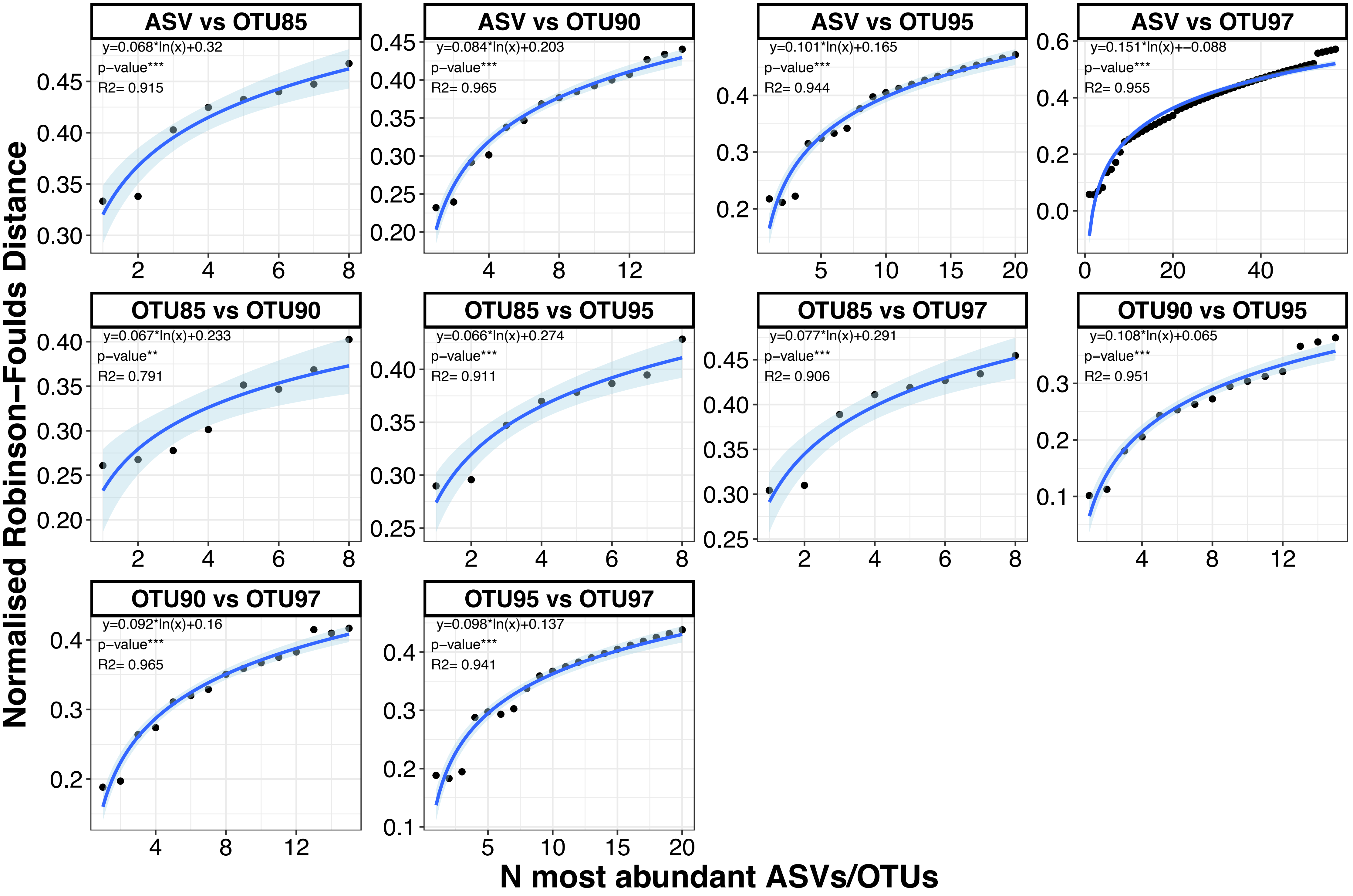

ASV vs OTU85
y=0.068*ln(x)+0.32
p−value***
R2= 0.915
ASV vs OTU90
y=0.084*ln(x)+0.203
p−value***
R2= 0.965
ASV vs OTU95
y=0.101*ln(x)+0.165
p−value***
R2= 0.944
ASV vs OTU97
y=0.151*ln(x)+−0.088
p−value***
R2= 0.955
OTU85 vs OTU90
y=0.067*ln(x)+0.233
p−value**
R2= 0.791
OTU85 vs OTU95
y=0.066*ln(x)+0.274
p−value***
R2= 0.911
OTU85 vs OTU97
y=0.077*ln(x)+0.291
p−value***
R2= 0.906
OTU90 vs OTU95
y=0.108*ln(x)+0.065
p−value***
R2= 0.951
OTU90 vs OTU97
y=0.092*ln(x)+0.16
p−value***
R2= 0.965
OTU95 vs OTU97
y=0.098*ln(x)+0.137
p−value***
R2= 0.941
Normalised Robinson−Foulds Distance
N most abundant ASVs/OTUs

2.C. *nirK*

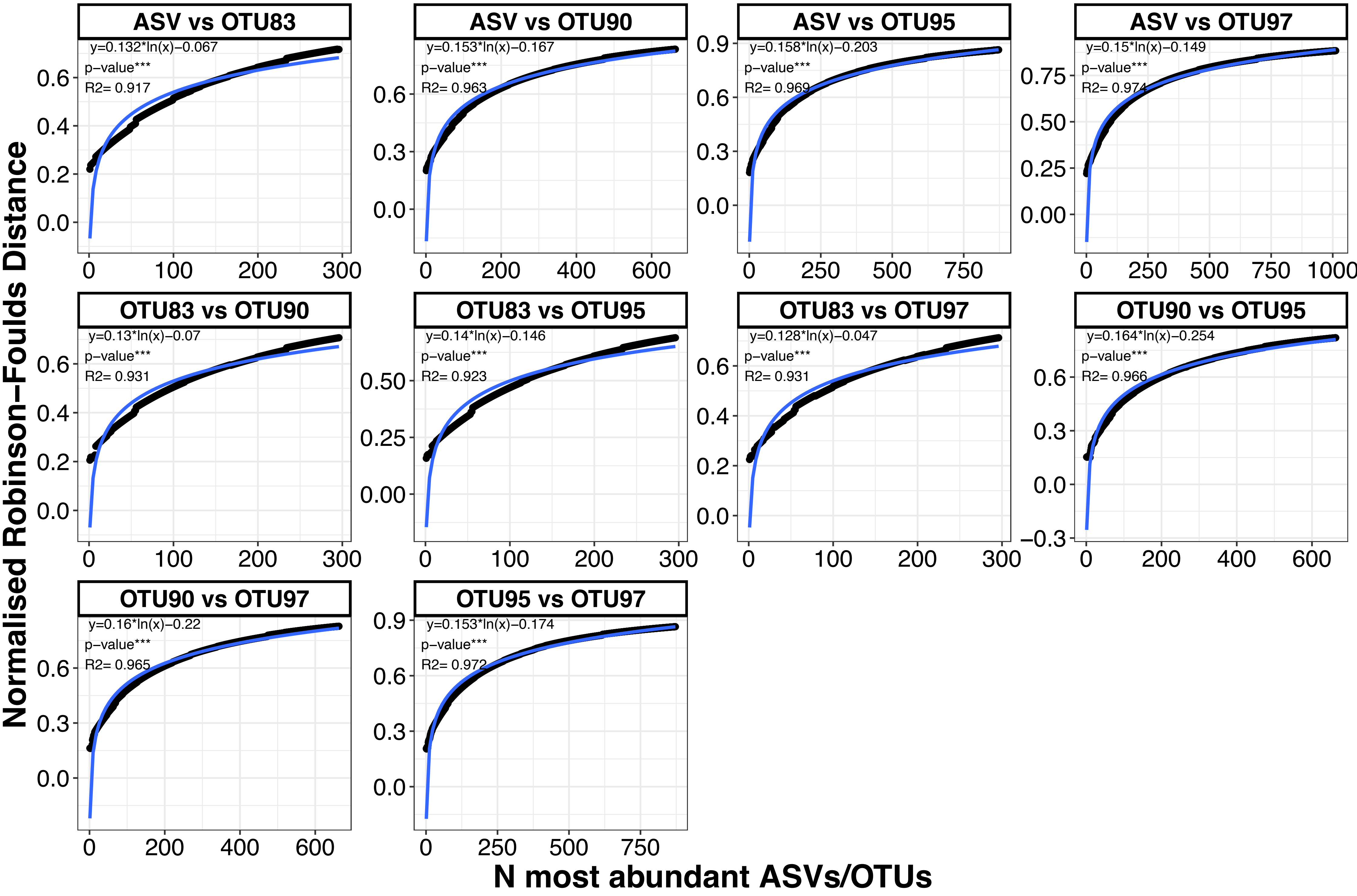

ASV vs OTU83
y=0.132*ln(x)−0.067
p−value***
R2= 0.917
ASV vs OTU90
y=0.153*ln(x)−0.167
p−value***
R2= 0.963
ASV vs OTU95
y=0.158*ln(x)−0.203
p−value***
R2= 0.969
ASV vs OTU97
y=0.15*ln(x)−0.149
p−value***
R2= 0.974
OTU83 vs OTU90
y=0.13*ln(x)−0.07
p−value***
R2= 0.931
OTU83 vs OTU95
y=0.14*ln(x)−0.146
p−value***
R2= 0.923
OTU83 vs OTU97
y=0.128*ln(x)−0.047
p−value***
R2= 0.931
OTU90 vs OTU95
y=0.164*ln(x)−0.254
p−value***
R2= 0.966
OTU90 vs OTU97
y=0.16*ln(x)−0.22
p−value***
R2= 0.965
OTU95 vs OTU97
y=0.153*ln(x)−0.174
p−value***
R2= 0.972
Normalised Robinson−Foulds Distance
N most abundant ASVs/OTUs

2.D. *nirS*

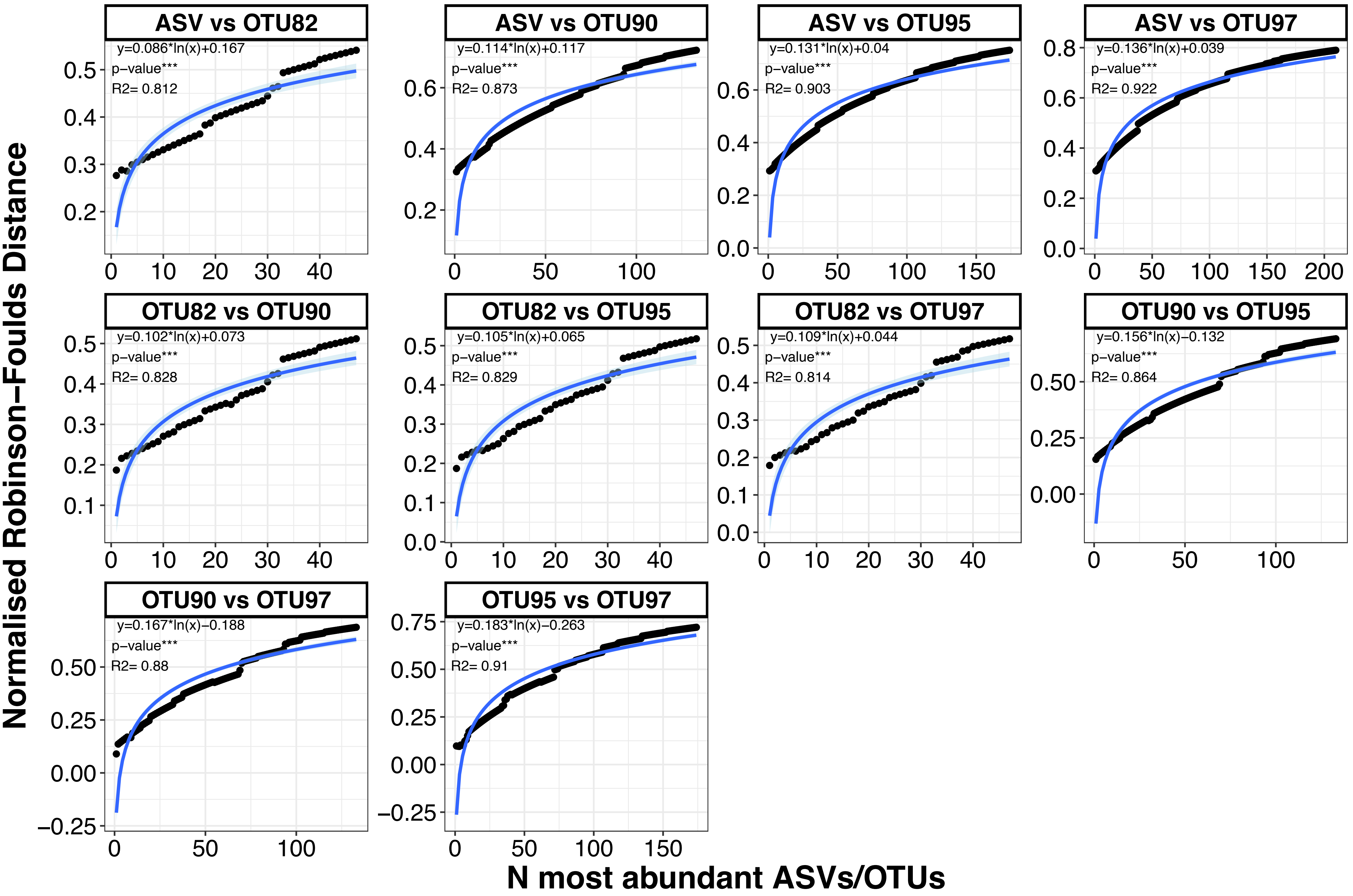

ASV vs OTU82
y=0.086*ln(x)+0.167
p−value***
R2= 0.812
ASV vs OTU90
y=0.114*ln(x)+0.117
p−value***
R2= 0.873
ASV vs OTU95
y=0.131*ln(x)+0.04
p−value***
R2= 0.903
ASV vs OTU97
y=0.136*ln(x)+0.039
p−value***
R2= 0.922
OTU82 vs OTU90
y=0.102*ln(x)+0.073
p−value***
R2= 0.828
OTU82 vs OTU95
y=0.105*ln(x)+0.065
p−value***
R2= 0.829
OTU82 vs OTU97
y=0.109*ln(x)+0.044
p−value***
R2= 0.814
OTU90 vs OTU95
y=0.156*ln(x)−0.132
p−value***
R2= 0.864
OTU90 vs OTU97
y=0.167*ln(x)−0.188
p−value***
R2= 0.88
OTU95 vs OTU97
y=0.183*ln(x)−0.263
p−value***
R2= 0.91
Normalised Robinson−Foulds Distance
N most abundant ASVs/OTUs

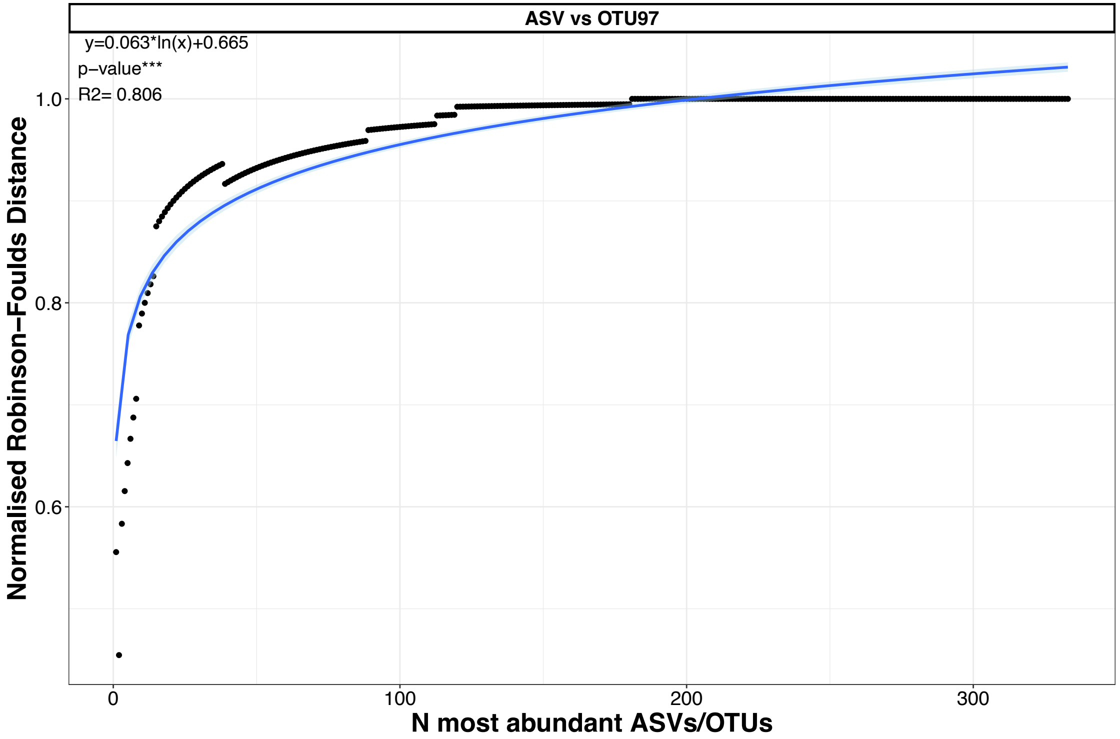

2.E. *nxrB*

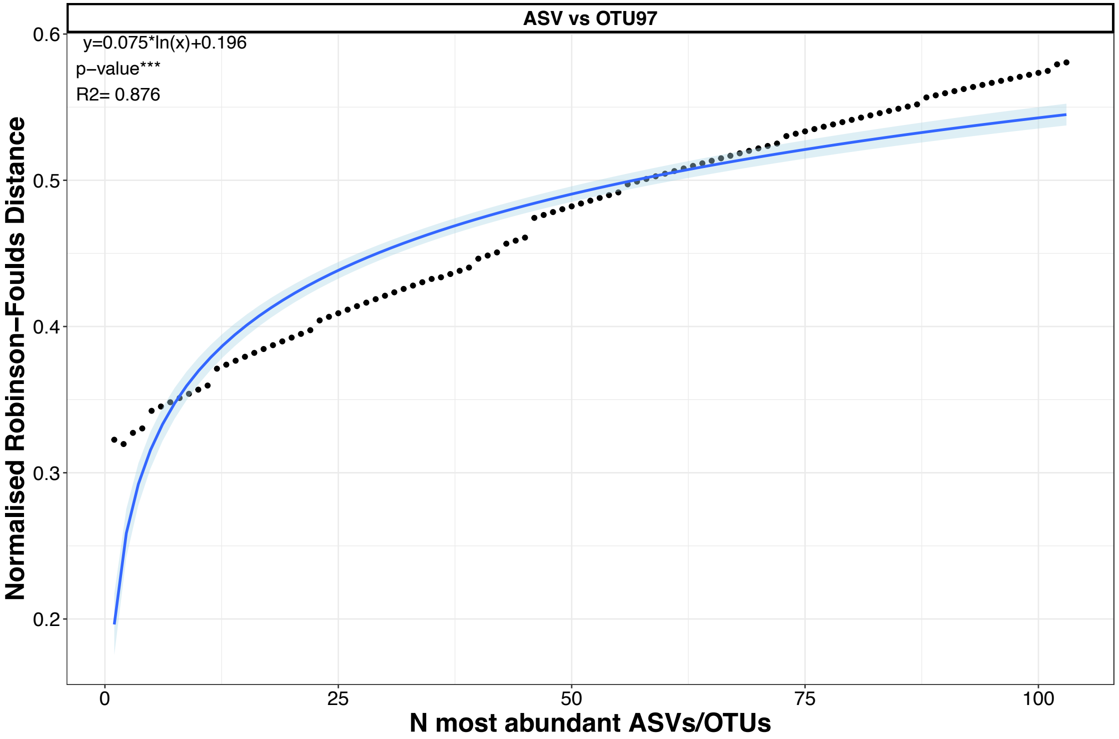

2.F. *nrfA*
