# Supplementary material for: Ecological Observations Based on Functional Gene Sequencing Are Sensitive to the Amplicon Processing Method
Source: mSphere. 2022 Aug 8;7(4):e00324-22. doi: 10.1128/msphere.00324-22 (PMC9429940; doi:10.1128/msphere.00324-22)
Supplement: TEXT S1 [file msphere.00324-22-s0004.docx]

**Supplementary Information**

**Supplementary Methods**

- **Biological and physio-chemical analysis**

*Nutrient analysis*

Nutrient content was determined on pore water extracted by centrifugation of the sediment samples and filtration through 0.2µm cellulose nitrate filters (samples diluted 1/10 for ammonia): ammonium, nitrite, nitrate, phosphate and silicates concentrations were determined using an autoanalyzer (Seal Analytical, GmbH Nordertedt, Germany) equipped with an XY-2 sampler. Salinity and pH were measured directly on unfiltered pore water.

*Density and Water content*

Density was determined by weighting a known volume of sample. Water content was determined by weight loss and after incubation at 60°C for 48h.

*TOC, DOC, DON and TN*

Sediment Total Organic Carbon (TOC) was determined by weight loss of dried samples incubated at 450°C for 12h. Pore water TOC, Dissolved Organic Carbon (DOC) and Total Nitrogen (TN) were measured on a Shimadzu TOC-L Analyzer. TOC was measured on unfiltered and DOC on filtered samples after acidification with HCl to pH below 2 and flushing with air for 5 minutes to remove inorganic carbon. Pore water total carbon/ total nitrogen ratio was calculated as (pore water TOC + pore water DOC)/ (pore water total nitrogen).

*Granulometry*

Sediment granulometry was measured on a Mastersizer laser granulometer 2000 equipped with a Hydro MU sampler. Clay was defined as particles with diameter smaller than 2µm, silt as particles with diameter between 2µm and 63µm and sand as particles with diameter higher than 63µm.

- **DNA extraction**

All surfaces and equipment were cleaned with 70% ethanol before sample processing. DNA was extracted based on the bead beating method of Griffiths et al (63). 0.5g of sediments were extracted using bead beating lysing tubes (Matrix tube E; MP Biomedical) and homogenised in 0.5ml CTAB/phosphate buffer (composition for 120 ml: 2.58g K_2_HPO_4_.3H_2_O; 0.10g KH_2_PO_4_; 5.0g CTAB; 2.05g NaCl) plus 0.5ml Phenol:Chlorophorm:Isoamyl alcohol (25:24:1 v:v:v). Lysis was carried out on the FastPrep system (MP Biomedical) (S: 6.0; 40sec) followed by a centrifugation at 12,000g for 20 min 4°C). The top aqueous layer was transferred to a fresh 1.5ml tube and mixed with 0.5ml chloroform:isoamyl alcohol (24:1 v:v). The mixture was centrifuged at 16,000g for 5 min (4°C) and the top aqueous layer was transferred to a new 1.5ml tube. DNA was precipitated by adding two volumes of a solution containing 30% poly(etlyleneglycol)_6000_ (PEG6000) and 1.6M NaCl for 2 hours on ice and subsequently recovered by centrifugation at 16,000 x g for 30 min (4°C). The pellet was washed with 1ml ice-cold 70% ethanol and centrifuged at 16,000g for 30 min (4°C). The ethanol wash was discarded, and the pellet was air dried and re-suspended in 40µl water. DNA preparations were stored at -80°C if not used immediately.

- **PCR and sequencing**

PCR amplifications were carried out using the HotStartTaq PCR kit (Qiagen) in the following mix 25 μl volume: 19.8 μl water, 0.5 μl of each primer (10 μM each), 0.5 μl dNTPs (10 μM each), 0.2 μl HotStartTaq, 2.5 μl of 10x PCR buffer and 1 μl DNA template (1/10 diluted). Primers used for sequencing were modified by adding Illumina adaptors at the 5’ end: 5’-TCG TCG GCA GCG TCA GAT GTG TAT AAG AGA CAG (forward adaptor); 5’-GTC TCG TGG GCT CGG AGA TGT GTA TAA GAG ACA G (reverse adaptor). A list of primers used to amplify nitrogen-cycle genes and the corresponding conditions is provided in Table 2. PCR amplicons were cleaned using the Agencourt AMPure XP beads (Beckman Coulter) following the manufacturer’s recommendations. Illumina indexes were attached using the Nextera XT Index Kit with the following PCR condition: 95°C-15min, (95°C-30sec, 55°C-30sec, 72°C-30sec) x 8 cycles and 72°C-5min. The resulting amplicons were purified using the Agencourt AMPure XP beads (Beckman Coulter) and eluted in 25µl water. After this step, some preparations were randomly chosen (two per gene target) and analysed on the Bioanalyser using the DNA 1000 Assay protocol (Agilent Technologies) to determine the average length of the amplicons and to check for the presence of unspecific products. Finally, DNA concentration was determined using fluorometric quantification method (Qubit) and molarity was calculated using the following equation:

(concentration in ng/μl) × 106 = (660 g/mol × average library size).

Libraries were pooled in equimolar amount and checked again on the Bioanalyser and the final library was sent to the Earlham Institute (Norwich Research Park, Norwich, UK) for Illumina MiSeq amplicon sequencing (300PE, 22 millions reads/ lane).

**Procedure for OTU construction**

- **Step 0: Organise the data**

for i in $(awk -F"_" '{print $1}' <(ls *.fastq) | sort | uniq); do mkdir $i; mkdir $i/Raw; mv $i*.fastq $i/Raw/.; done

After this step, for each sample we will have a folder with the mane of the sample and within it a ‘Raw’ folder containing the raw forward and reverse reads. This step needs to be repeated for each target genes, which will be in a separate folder. For example, we have AOB in the folder located at “/home/analysis/AOB/”.

Next, set the variable d the directory where the sequences are held. In this example, d=“/home/analysis/AOB/”

- **Step 1: Quality trimming using sickle**

for i in $(ls -d *); do cd $i;cd Raw; R1=$(ls *_R1_*.fastq); R2=$(ls *_R2_*.fastq); cd .. ; sickle pe -f Raw/$R1 -r Raw/$R2 -o ${R1%.*}_trim.fastq -p ${R2%.*}_trim.fastq -s ${R1%.*}_singlet.fastq -q 20 -l 50 -t "sanger";cd ..; done

- **Step 2: Create a folder with the corrected reads using Spades**

for i in $(ls */ -d *); do cd $i; /home/opt/SPAdes-3.13.0-Linux/bin/spades.py -1 *_R1_*trim.fastq -2 *_R2_*trim.fastq -o . --only-error-correction --careful --disable-gzip-output ; cd ..; done

- **Step 3: Overlap reads using Pandaseq**

export PATH=/home/opt/miniconda5/bin:$PATH

source activate pandaseq

for i in $(ls */ -d); do cd $i; awk 'NR % 4==1{$0=$0" 1:N:0:GGACTCCTGTAAGGAG"}1' corrected/*R1*.cor.fastq > corrected/forward_corrected.fastq; awk 'NR % 4==1{$0=$0" 2:N:0:GGACTCCTGTAAGGAG"}1' corrected/*R2*.cor.fastq > corrected/reverse_corrected.fastq; pandaseq -f corrected/forward_corrected.fastq -r corrected/reverse_corrected.fastq -B -d bfsrk -A rdp_mle -o 10 > $(basename ${i})".overlap.fasta"; cd ..; done

- **Step 4: Combine the overlap reads in a VSEARCH format**

cd ..

mkdir AOB_VSEARCH

cd AOB_VSEARCH

for i in $(ls -d ../AOB/*/); do awk -v k=$(basename ${i}) '/^>/{$0=">barcodelabel="k";S"(++i)}1' < $i/*.overlap.fasta; done > multiplexed.fasta

- **Step 5: Linearise fasta file**

awk 'NR==1 {print ; next} {printf /^>/ ? "\n"$0"\n" : $1} END {print}' multiplexed.fasta > multiplexed_linearized.fasta

- **Step 6: Dereplicate, sort and get rid of singletons**

/home/opt/vsearch/bin/vsearch --threads 20 --derep_fulllength multiplexed_linearized.fasta --minuniquesize 2 --sizein --sizeout --fasta_width 0 --uc multiplexed_linearized_dereplicated_vsearch_min2.uc --output multiplexed_linearized_dereplicated_vsearch_min2.fasta

- **Step 7: Cluster at 0.97% identity (change percentage identity here)**

/home/opt/vsearch/bin/vsearch --threads 20 --cluster_size multiplexed_linearized_dereplicated_vsearch_min2.fasta --id 0.97 --strand both --sizein --sizeout --fasta_width 0 --uc multiplexed_linearized_dereplicated_vsearch_min2_preclustered.uc --centroids multiplexed_linearized_dereplicated_vsearch_min2_preclustered.fasta

- **Step 8: Denovo chimera removal step**

/home/opt/vsearch/bin/vsearch --threads 20 --uchime_denovo multiplexed_linearized_dereplicated_vsearch_min2_preclustered.fasta --sizein --sizeout --fasta_width 0 --nonchimeras multiplexed_linearized_dereplicated_vsearch_min2_preclustered_nonchimeras.fasta

- **Step 9: Reference based chimera removal step**

/home/opt/vsearch/bin/vsearch --threads 20 --uchime_ref multiplexed_linearized_dereplicated_vsearch_min2_preclustered_nonchimeras.fasta --db /home/opt/vsearch_GOLD_DATABASE/gold.fasta --sizein --sizeout --fasta_width 0 --nonchimeras multiplexed_linearized_dereplicated_vsearch_min2_preclustered_nonchimeras_ref.fasta

- **Step 10: Rename the OTUs**

/home/opt/vsearch/bin/vsearch --threads 20 --fastx_filter multiplexed_linearized_dereplicated_vsearch_min2_preclustered_nonchimeras.fasta --threads 20 --sizein --sizeout --fasta_width 0 --relabel OTU_ --fastaout otus.fa

awk '/^>/{gsub(";.*","",$0)}1' otus.fa > otus_backup; mv otus_backup otus.fa

- **Step 11: Match reads against the OTUs**

/home/opt/vsearch/bin/vsearch --threads 20 --usearch_global multiplexed.fasta --threads 20 --db otus.fa --id 0.97 --sizein --sizeout --fasta_width 0 --qmask none -dbmask none --otutabout otu_table.txt

- **Step 12: Convert to upper case**

bioawk -cfastx '{print ">"$name"\n"toupper($seq)}' otus.fa > otus_upper.fa

- **Step 13: Enable Qiime2 and generate a phylogenetic tree**

export PATH=/home/opt/miniconda2/bin:$PATH

source activate qiime2-2019.7

qiime tools import --type 'FeatureData[Sequence]' --input-path otus_upper.fa --output-path otus.qza

unset MAFFT_BINARIES

qiime phylogeny align-to-tree-mafft-fasttree --i-sequences otus.qza --o-alignment aligned-otus.qza --o-masked-alignment masked-aligned-otus.qza --p-n-threads 0 --o-tree unrooted-tree.qza --o-rooted-tree rooted-tree.qza

qiime tools export --input-path rooted-tree.qza --output-path output

**R scripts for ASV construction:**

This script is based on the tutorial found at <https://benjjneb.github.io/dada2/tutorial.html>. To run this script, first store all samples (forward and reverse reads) in the same folder and generate a txt file with the name of each sample per rows.

- **Step 1: Load the required libraries**

library(Rcpp)

library(dada2)

- **Step 2: set variables**

samples <- scan("samples.txt", what="character")

forward_reads <- paste0(samples, "_R1.fastq")

reverse_reads <- paste0(samples, "_R2.fastq")

filtered_forward_reads <- paste0(samples, "_R1_filtered.fastq")

filtered_reverse_reads <- paste0(samples, "_R2_filtered.fastq")

- **Step 3: Quality filtering the value for trimRight and trimleft are to be adjusted for each genes as listed in Supplementary Table 1**

filtered_out<-filterAndTrim(forward_reads, filtered_forward_reads,reverse_reads, filtered_reverse_reads,

rm.phix=TRUE, trimRight = c(,), trimLeft = c(,))

- **Step 4: generate an error model**

err_forward_reads <- learnErrors(filtered_forward_reads)

err_reverse_reads <- learnErrors(filtered_reverse_reads)

- **Step 5: dereplication**

derep_forward <- derepFastq(filtered_forward_reads, verbose=TRUE)

names(derep_forward) <- samples

derep_reverse <- derepFastq(filtered_reverse_reads, verbose=TRUE)

names(derep_reverse) <- samples

- **Step 6: infer ASVs:**

dada_forward<-dada(derep_forward,err=err_forward_reads,pool="pseudo")

dada_reverse<-dada(derep_reverse,err=err_reverse_reads,pool="pseudo")

- **Step 7: Merge forward and reverse reads**

merged_amplicons<-mergePairs(dada_forward,derep_forward,dada_reverse,derep_reverse, trimOverhang=FALSE,minOverlap=)

- **Step 8: Remove chimeras**

seqtab.nochim <- removeBimeraDenovo(seqtab, verbose=T)

- **Step 9: Change the name of the ASVs**

asv_seqs <- colnames(seqtab.nochim)

asv_headers <- vector(dim(seqtab.nochim)[2], mode="character")

for (i in 1:dim(seqtab.nochim)[2]){asv_headers[i] <- paste(">ASV", i, sep="_")}

- **Step 10: Generate a count table and rep seqs fasta file**

asv_fasta <- c(rbind(asv_headers, asv_seqs))

for (i in 1:length(asv_fasta)){asv_fasta [[i]]=toupper(asv_fasta [[i]])}

write.fasta(sequences=asv_fasta,names = names(asv_fasta), file.out=paste("ASVs.fasta"))

asv_tab <- t(seqtab.nochim)

row.names(asv_tab) <- sub(">", "", asv_headers)

write.table(asv_tab, "ASVs_counts.tsv", sep="\t", quote=F, col.names=NA)

- **Step 11: Generate a summary table**

sum(seqtab.nochim)/sum(seqtab)

getN <- function(x) sum(getUniques(x))

summary_tab<-data.frame(row.names=samples, dada2_input=filtered_out[,1],filtered=filtered_out[,2], dada_f=sapply(dada_forward, getN),dada_r=sapply(dada_reverse, getN), merged=sapply(merged_amplicons, getN),nonchim=rowSums(seqtab.nochim))

summary_final=data.frame(

dada2_input=sum(summary_tab$dada2_input),

filtered=sum(summary_tab$filtered),

dada_f=sum(summary_tab$dada_f),

dada_r=sum(summary_tab$dada_r),

merged=sum(summary_tab$merged),

nonchim=sum(summary_tab$nonchim))

summary_final$final_perc_reads_retained=round(summary_final$nonchim/summary_final$filtered*100, 1)

write.csv(summary_final,file ='Summary table AOA ASV.csv')

- **Step 12: Generate a phylogenetic tree (on server using Qiime2)**

export PATH=/home/opt/miniconda2/bin:$PATH

source activate qiime2-2019.7

qiime tools import --type 'FeatureData[Sequence]' --input-path ASV.fasta --output-path ASV.qza

unset MAFFT_BINARIES

qiime phylogeny align-to-tree-mafft-fasttree --i-sequences ASV.qza --o-alignment aligned-rep-seqs.qza --o-masked-alignment masked-aligned-rep-seqs.qza --p-n-threads 0 --o-tree unrooted-tree.qza --o-rooted-tree rooted-tree.qza

qiime tools export --input-path rooted-tree.qza --output-path output

**R scripts for OTU/ASV quality check:**

- **Step 1: Load the required libraries**

library(seqinr)

library(ggplot2)

library(scales)

- **Step 2: Set the ‘length’ and ‘frame’ variables; for OTU, we’ll also need the ‘fw’ and ‘rev’ variables to remove primer sequences**

if(target=="AOA"){length=221;frame=1;fw=19;rev=20}

if(target=="AOB"){length=452;frame=2;fw=18;rev=21}

if(target=="nirK"){length=436;frame=1;fw=17;rev=20}

if(target=="nrfA"){length=223;frame=1;fw=17;rev=17}

if(target=="nirS"){length=224;frame=2;fw=18;rev=18}

if(target=="nxrB"){length=453;frame=2;fw=16;rev=16}

- **Step 3: load the fasta file containing the representative sequences; for OTUs, remove the primer sequences (in the case of ASVs, the primers are already removed in the processing step)**

#For OTU tables:

seqs<-read.fasta(<INSERT PATH TO OTU SEQUENCES HERE>, as.string = F)

for(i in 1:length(seqs)){seqs[[i]]=seqs[[i]][(fw+1):(length(seqs[[i]])-rev)]}#to remove primer sequences for OTUs

abund_table=read.delim(<INSERT PATH TO OTU ABUNDANCE TABLE HERE>,h=T,row.names = 1)

#For ASV tables:

seqs<read.fasta(<INSERT PATH TO ASV SEQUENCES HERE>, as.string = F)

abund_table=read.delim(<INSERT PATH TO ASV ABUNDANCE TABLE HERE>,h=T,row.names = 1)

- **Step 4: Translate DNA sequences to amino acid and remove sequences that contain STOP codons**

FRAME=frame

STOP=c()

j=1

for (i in 1:length(seqs)){

translate=translate(seqs[[i]], frame = FRAME)

write.fasta(sequences = translate, names = names(seqs)[i], file.out = paste(target,method,percent,"AA_RL.fasta", sep="_"), open="a")

if('*'%in%translate){STOP[j]=i;j=j+1}

}

AA=read.fasta(file=paste(target,method,percent,"AA_RL.fasta",sep="_"), seqtype = "AA", as.string = T)

AA_curated=AA[-STOP]

- **Step 5: find a list of unique proteins to be blasted**

Prot_check=NULL

for (i in 1:length(AA_curated)){

tmp=data.frame(

Unique=attr((AA_curated[AA_curated %in% AA_curated[i]]),"names")[1],

Multiple=paste(attr((AA_curated[AA_curated %in% AA_curated[i]]),"names"),collapse = ';')

)

if(i==1){Prot_check=tmp}else{Prot_check=rbind(Prot_check,tmp)}

print(paste(round((i/length(AA_curated)*100),2),'%',sep=''))

}

library(dplyr)

Prot_check=distinct(Prot_check,.keep_all = FALSE)

Prot.to.blast=AA_curated[Prot_check$Unique]

- **Step 5: generate a file containing the proteins with the wrong length to be checked**

if(target=="AOB"){length_AA=150}

if(target=="AOA"){length_AA=73}

if(target=="nirS"){length_AA=74}

if(target=="nirK"){length_AA=145}

if(target=="nxrB"){length_AA=150}

if(target=="nrfA"){length_AA=74}

seqs.Wrong.length=vector(mode = "character")

seqs.Right.length=vector(mode = "character")

for (i in 1:length(Prot.to.blast)){

if(nchar(Prot.to.blast[[i]]) !=length_AA ){

seqs.Wrong.length[i]=attr(Prot.to.blast[i],"names")}else(seqs.Right.length[i]=attr(Prot.to.blast[i],"names"))

seqs.Wrong.length=seqs.Wrong.length[!is.na(seqs.Wrong.length)]

seqs.Right.length=seqs.Right.length[!is.na(seqs.Right.length)]

}

Wrong.seqs=Prot.to.blast[seqs.Wrong.length]

Right.seqs=Prot.to.blast[seqs.Right.length]

write.fasta(sequences = Wrong.seqs, names = names(Wrong.seqs), file.out = paste(target,method,percent,"AA_WL_to_check.fasta",sep='_'))

- **Step 6: BLAST the sequences generated in the previous step**

**The previous step will create a fasta file containing protein sequences that do not match the expected length of the target gene. These sequences will be BLASTed using the BLASTp algorithm (against the standard nr database); After BLASTp, generate a vector in R containing the list of sequences that do match with the target enzyme:**

list=c(<INSERT LIST OF SEQUENCES HERE>)

- **Step 7: Generate a list of ‘correct’ sequences and print fasta files**

correct.prots=c(unlist(strsplit(Prot_check[Prot_check$Unique%in%names(Right.seqs),'Multiple'],split=';')),unlist(strsplit(Prot_check[Prot_check$Unique%in%list,'Multiple'],split=';')))

final_AA_corrected=AA_curated[correct.prots]

write.fasta(sequences = final_AA_corrected, names = names(final_AA_corrected), file.out = paste(<INSERT NAME OF PROTEIN SEQUENCES FILE HERE>)))

final_corrected=seqs[correct.prots]

write.fasta(sequences = final_corrected, names = names(final_corrected), file.out = paste(<INSERT NAME OF DNA SEQUENCES FILE HERE>))

- **Step 8: at the beginning of each scripts for downstream statistical analyses, curate the abundance table and phylogenetic trees by retaining only the correct sequences**

AA=read.fasta(<INSERT PATH TO THE PROTEIN FASTA FILE GENERATED IN STEP 7 HERE>, seqtype = "AA", as.string = T)

#curation of the abundance table:

abund_table<-abund_table[,(colnames(abund_table) %in% names(AA))]

# After curation of the abundance table, trim the phylogenetic tree:

tree$tip.label=gsub("'","",tree$tip.label)

tree=keep.tip(tree, colnames(abund_table
