# Supplementary material for: Ecological Observations Based on Functional Gene Sequencing Are Sensitive to the Amplicon Processing Method
Source: mSphere. 2022 Aug 8;7(4):e00324-22. doi: 10.1128/msphere.00324-22 (PMC9429940; doi:10.1128/msphere.00324-22)
Supplement: TABLE S1 [file msphere.00324-22-s0005.docx]

**Supplementary Table 1: Parameters for the ASV quality filtering**

|  | trimRight(c(,)) | | trimLeft(c(,)) | |
| --- | --- | --- | --- | --- |
|  | Forward | Reverse | Forward | reverse |
| AOA *amoA* | 60 | 100 | 19 | 20 |
| AOB *amoA* | 25 | 70 | 18 | 21 |
| *nxrB* | 25 | 85 | 16 | 16 |
| *nirS* | 60 | 100 | 18 | 18 |
| *nirK* | 25 | 85 | 17 | 20 |
| *nrfA* | 60 | 100 | 17 | 17 |
