# Supplementary material for: Ecological Observations Based on Functional Gene Sequencing Are Sensitive to the Amplicon Processing Method
Source: mSphere. 2022 Aug 8;7(4):e00324-22. doi: 10.1128/msphere.00324-22 (PMC9429940; doi:10.1128/msphere.00324-22)
Supplement: TABLE S2 [file msphere.00324-22-s0006.docx]

**Supplementary Table 2: Expected length for the target genes.** The excepted length for doth the nucleotide and amino acid (AA) sequences is calculated for the amplicon without primer sequences.

|  | Expected length | |
| --- | --- | --- |
|  | Nucleotide sequences | AA sequences |
| AOA *amoA* | 221 | 73 |
| AOB *amoA* | 452 | 150 |
| *nxrB* | 453 | 150 |
| *nirS* | 224 | 74 |
| *nirK* | 436 | 145 |
| *nrfA* | 223 | 74 |
