# Supplementary material for: Ecological Observations Based on Functional Gene Sequencing Are Sensitive to the Amplicon Processing Method
Source: mSphere. 2022 Aug 8;7(4):e00324-22. doi: 10.1128/msphere.00324-22 (PMC9429940; doi:10.1128/msphere.00324-22)
Supplement: TABLE S3 [file msphere.00324-22-s0007.docx]

**Supplementary Table 3: List of Reference sequences for phylogenetic trees.**

| **Target gene** | **Reference sequence** |
| --- | --- |
| **AOA *amoA*** | CP010868;JOSY01000059;JOTD01000072;JOKN01000008;EU2399591;CP000866;LIAL01000053;LIAK01000316;CP021324;MG5525151;KX950755;KF957666;AEXL02000009;AB546962;CP011070;KR737580;MG5524901;AEGP01000066;JQ345897;JQ345873;JQ345876;JQ345805;JQ345880;JQ345885;JQ345886;JQ345869;MG5524941;JQ345894;HQ3311171;HM345608;HM345610;AB4270541;JN2274891;LN890280;MG5525271;DQ1487741;HM345609;MG5525671;MG5525041;MG5525411;JN4181781;JX4262781;KJ6453201;MG5525351;MG5525431;KC5593471;MG5525561;AJ6274221;JX0108061;KC5593641;FR7731591;FR773159;CP007536;KC5592941;EU281321;CP0024081;CP002408;EU281320;EU281318;EU281319;EU281317;MG5525061;MG5525611;MG5525211;KM1169121;HM7546561;MG5525501;MG5525381;EU6713951;MG5525591;EU9252861;MG5525441;DQ1489021;KF6687741;EU3394281;JF4305591;MG5525581;AACY014359671;CP007026;CP011097;KP027213;KJ1584581;CP003843 |
| **AOB *amoA*** | U76553;AJ238544.1;AY123825.1;AJ298717.1;AJ298719.1;U91603;FNVK01000006;CP000103;U15733;CP038033.1;X90822;AJ298702;Y123817.1;AJ298689.1;AF042171.1;AY352922.1;Z97858.1;AJ298697.1;KC477403.1;AY123823.1;AY123834.1;AJ298698.1;AY249743.1;AJ298722.1;AY123836.1;AY123839.1;AJ388584.1;AF272403.1;AJ388585.1;AF272406.1;AJ388587.1;AF272405.1;AJ388586.1;AF272400.1;AF339042;AF272404.1;AJ238495.1;AF272399.1;AF272398.1;AJ238541.1;AB070981.1;JN099309.1;U31649;AJ298710.1;AL954747;AL954747;U72670;U51630;AJ298713;CP000450;AF272521.1;CP001798;CP002086.1;AF153344;U96611.1;AF047705.1;AF508998.1;AF509001;CP000127.1;AF272402.1;AF314753;AY702580.1;KF970501.1;AY736940.1;EU244559.1;KC735890.1;GU453824.1;KF970608.1;EF222057.1;EU651496.1 |
| ***nirK*** | CP002977;CP000507;AAXY01000002;EF016120;AF339045;CP002552;JPGN01000005;CP021060;AEQP01000008;ACKY01000043;AGCM01000121;AY072264;CP002026;EF202175;AM230813;EF363542;AKMR01000006;FN555529;CP002663;AFRQ01000082;HM060299;AEVM01000035;CP000463;CP000390;AJXZ01000049;AGSN01000255;FN600573;AAMT01000003;U62291;FN600574;EF363545;FJ598613;Z48635;AM403570;CP013964;ACOR01000007;LPQX01000062;EF363544;AM230830;EF363543;AF083948;CP000138;CP001807;CP003372;FN555205;AJ278286;CP002344;AP006840;CP001337;AFNV01000066;CP000529;CP000103;AGSL01000063;JUEC02000103;FQ311871;CAHT01000030;CP000774;CP002083;CP002008;CP000747;CP001823;ABVL01000009;CP020946;FQ312002;AJSX01000022;CP000746;AE016827;CP002667;AJGB01000025;AEWV01000013;ATRF01000062;AFWR01000032;EU339313;AFHU01000052;FUKV01000029;CP001707;AGSC01000047;CP003280;AJJU01000017;CP002349;CP003281;CP000777;CP000786;CP002959;CP001280;CP001672;AP012305;CP003171;AL646053;CP002446;AE016825;AAOF01000033;ACYE01000146;AP007161;DQ390349;DQ384327;GU233007;CP000481;AEPP01000034;ACYT02000064;ACGD01000001;ACLI01000031;AFQC01000053;CP000088;CP001630;AP012319;FMHX01000003;CP015224;LZZG01000012;AGJG01000004;CP001727;CP002050;AKKV01000036;AP010904;AANZ01000003;CP003379;CP000934;CP002355;AHJE01000003;ABLD01000001;ABLC01000001;CP001193;AAPJ01000003;AATP01000012;LC066397;AM049177;AKFK01000061;CP001016;AKFK01000061;CP001510;FP103042;CP000495;LJIC01000107;CP000322;CP001196;ADVZ01000001;AP012206;CP000539;CP001220;CP001674;CP000284;CP002056;CP002745;CU207211;CP013136;AEPR01000232;CP002021;CP002959;BAFH01000003;CT573071;ABOX02000056;CP000113;CP003389;AP009153;CP000449;AM746676;FR872581;AFCE01000055;AFHT01000094;ACLW01000047;CKVR01000203;AFRV01000005;CP000154;ACLW01000047;AKKV01000036;CP003125;LUCT01000050;LQYU01000036;AP008955;CP001823;CP000358;ADVZ01000001;CP000699;ACWO01000117;FN649414;CP002454;AY596295;CP001956;AM774415;AGIR01000001;CP001365;CP002839;CP002630;CP000389;MJVD01000090;AP006619;CP000249;CP000386;FP929003;FP929003;CP000115;CP000961;CP000975;CU207211;CP000699;CP000571;CP005964;CP012712;LQYU01000017;CP000866;AEXL02000161;AEGP01000037;AFPU01000001;AEXL02000051;AFPU01000001;CP000875;GQ226037;LJJR01000007;AFCE01000115;CP001905;AAMV01000015;AAYC01000016 |
| ***nirS*** | CP012154;AM492191;AXCA01000197;AUNC01000001;ANHY01000019;CP000830;AXCE01000003;CP000032;CP002623;AONI01000011;AUNB01000023;LJSU01000028;ACYY01000001;CP002568;LN997848;LJYW01000001;AP012603;AB480490;CP012918;FN555562;CP001715;AM902716;AJ224913;FN555559;AM269907;AP012029;CP000804;LGKO01000004;LGHJ01000006;CP001807;AP011112;CP001230;CP013355;CP001130;CP001080;AP009178;ABHJ01000010;CT573071;LVEI02000001;CP002361;CP002778;CP003249;CP001130;CP001230;CP001080;AP009178;LCJQ01000011;CP002205;CP000153;AP009179;CP011308;CP002452;AE009441;LGCM01000014;CYTK01000001;CYTB01000003;APJX01000019;LUUJ01000129;GQ241349;CP019285;AOMA01000203;AFOC01000069;AFZB01000041;LWGJ01001372;JYNI01000072;FO203512;CP000083;LKBA01000024;CXTY01000013;CP003147;LMCB01000159;LXTQ01000001;FLQY01000367;AP012547;CP000091;AB480487;X91394;CP006668;LT607802;DQ865925;DQ865926;FN555563;ACIS01000002;LDUI01000036;CYHA01000002;CP001965;CBTJ020000111;FN555558;BAFJ01000002;LODL01000010;AMXA01000010;CP003153;CYHH01000010;LMFP01000027;CP013692;LKCX01000031;ACQT01000010;LVWD01000003;JSYI01000121;CP002657;LMHQ01000004;AP012304;CR555306;LARU01000002;AP014879;AP014936;AJ401462;AVCJ01000012;CP003989;CYHB01000003;CP001707;CP000155;AB092344;FKIY01000075;GU122964;LLUR01000051;AB744658;CLJU01000018;LDUG01000028;CP016268;KC855765;ANIE01000004;AP014633;LUTY01001461;ABBZ01001128;CP011971 |
| ***nrfA*** | CP001998;AGSB01000070;CP002546;BX294152;ANOF01000030;CP011270;LSTD01000068;ABCE01000055;CP000769;AGFC01000020;CP003152;CP007656;LUKD01000009;AUNE01000029;BAHD01000004;LN885086;LSTJ01000064;ABVL01000003;CP012332;JEME01001587;JEMA01001196;CP001032;BAFH01000002;BAFN01000001;ADVR01000012;CP002042;CP002343;ACVN02000219;CP014228;CP002734;AKFT01000013;CP002045;LGYI01000008;BAFE01000053;BAGZ01000017;CP007053;ABEA02000009;CTBL01000057;AYUF01000499;ACZK01000016;ACZS01000009;ABCK01000003;CP003349;CP013118;CP007451;BAMD01000001;LGIA01000045;ATNM01000109;CP003346;JMIH01000004;AWXR01000059;CP003561;LCTZ01000002;BAZW01000025;CP003557;CP003418;LN515532;AUTU01000024;CP000140;ACUD01000012;CAZK010000075;AE015928;CYZB01000009;CP003249;LN890655;AP012337;CYUD01000001;ADGM01000025;AP012344;LGKO01000006;LGHJ01000015;AP012029;LKCM01000100;LGCM01000018;ACJM01000021;AGJQ01000001;CP001785;CP002770;LGFP01000002;CP001147;MAVV01000037;CP000478;APJX01000004;CP001336;AGJA01000003;LWLG01000001;CP011232;ASSY01000010;LQAQ01000093;CP014227;CP009687;CP000853;CP000724;JYHU01000012;AXUN02000001;CP001708;AEXM01000028;CP001684;ACUX02000019;CP001682;FP929047;AP013105;ASSY01000001;FCNB01000136;ADLX01000075;AVKQ01000148;ADMF01000009;AFBP01000004;CP015403;LDXO01000001;CP000482;CP009788;AP014945;LJUR01000188;AGFB01000001;CP001940;CP002347;FTRD01000035;CP015403;ATCF01000018;ACJM01000009;CP001899;LJNM01000320;LAKY01000063;AP006840;CP000930;CP000141;CP000612;CP000448;CP001791;CP012152;CP012602;AJLR01000037;CP001197;CP001629;ACJN02000001;CP002432;AGFD01000039;CP007268;CP000544;ACKP02000048;CP002637;CYYU01000009;AFHQ01000042;CP000252;CP010978;ASXP01000006;AKVO01000077;AAWL01000044;CTRP01000005;AFGF01000173;CP002028;ADLW01000010;CP006772;CP001649;AAEW02000013;CP010311;CP000821;CP002209;CP002209;ABCQ01000051;LOCN01000019;LASY01000106;ANFM02000018;ACZT01000020;LN554846;CR378667;LRRV01000001;LT575468;CP004141;CP023706;CP011254;CP007044;LGYB01000009;ABXW01000073;LDVW01000103;BAHA01000047;JZUW01000045;AFCV01001456;LNHZ01000054;LBDB01000001;LJET01000120;AKMX01000086;CP000266;AXTI01000040;LN832404;CBWF010001313;LDAX01000015;CYUC01000002;JNGI01000021;BAFF01000004;CP001657;ACCD01000001;FR729477;CP010423;JTJR01000035;AE016827;CBMK010000015;CP008918;CP004391;FCYU01000002;L42023;AJSV01000029;AEPS01000002;CP001607;CP013830;MLAB01000030;LFYE01000008;CP007715;ADOG01000002;CP006954;JWIZ01000035;ABKM01000020;CP015099;AXZL01000076;CP007201;BX571659;CR522870;LJTL01000139;JMIY01000007 |
| ***nxrB*** | KC884897;LN885086;CP011801;JZQY01000048;FP929003;KC884861;KC884904; LSTC01000028; KC884871; CZQA01000001; CZPZ01000019; KC884903 |
