# Supplementary material for: Ecological Observations Based on Functional Gene Sequencing Are Sensitive to the Amplicon Processing Method
Source: mSphere. 2022 Aug 8;7(4):e00324-22. doi: 10.1128/msphere.00324-22 (PMC9429940; doi:10.1128/msphere.00324-22)
Supplement: TABLE S4 [file msphere.00324-22-s0008.docx]

**Supplementary Table 4: Effect of amplicon reconstruction method on alpha diversity measures.** Post-hoc test were carried out using the TukeyHSD () function in R.

| **Index** | **Target** | **Effect** | **Comparison** | **p.value** |
| --- | --- | --- | --- | --- |
| Richness | AOB | PercentageID | 90-85 | 0.22271388 |
| Richness | AOB | PercentageID | 95-85 | 4.82E-09 |
| Richness | AOB | PercentageID | 97-85 | 0 |
| Richness | AOB | PercentageID | ASV-85 | 0 |
| Richness | AOB | PercentageID | 95-90 | 3.65E-05 |
| Richness | AOB | PercentageID | 97-90 | 0 |
| Richness | AOB | PercentageID | ASV-90 | 0 |
| Richness | AOB | PercentageID | 97-95 | 0 |
| Richness | AOB | PercentageID | ASV-95 | 0 |
| Richness | AOB | PercentageID | ASV-97 | 0 |
| Richness | AOB | Type | Runnel-Ridge | 0.80171503 |
| Shannon | AOB | PercentageID | 90-85 | 0.00340046 |
| Shannon | AOB | PercentageID | 95-85 | 0 |
| Shannon | AOB | PercentageID | 97-85 | 0 |
| Shannon | AOB | PercentageID | ASV-85 | 0 |
| Shannon | AOB | PercentageID | 95-90 | 0 |
| Shannon | AOB | PercentageID | 97-90 | 0 |
| Shannon | AOB | PercentageID | ASV-90 | 0 |
| Shannon | AOB | PercentageID | 97-95 | 0 |
| Shannon | AOB | PercentageID | ASV-95 | 0 |
| Shannon | AOB | PercentageID | ASV-97 | 0 |
| Shannon | AOB | Type | Runnel-Ridge | 0.125425 |
| Simpson | AOB | PercentageID | 90-85 | 0.06327342 |
| Simpson | AOB | PercentageID | 95-85 | 0 |
| Simpson | AOB | PercentageID | 97-85 | 0 |
| Simpson | AOB | PercentageID | ASV-85 | 0 |
| Simpson | AOB | PercentageID | 95-90 | 0 |
| Simpson | AOB | PercentageID | 97-90 | 0 |
| Simpson | AOB | PercentageID | ASV-90 | 0 |
| Simpson | AOB | PercentageID | 97-95 | 0.09839341 |
| Simpson | AOB | PercentageID | ASV-95 | 1.55E-07 |
| Simpson | AOB | PercentageID | ASV-97 | 0.00269602 |
| Simpson | AOB | Type | Runnel-Ridge | 0.07717549 |
| Richness | AOA | PercentageID | 90-85 | 0.49773782 |
| Richness | AOA | PercentageID | 95-85 | 7.34E-08 |
| Richness | AOA | PercentageID | 97-85 | 0 |
| Richness | AOA | PercentageID | ASV-85 | 0 |
| Richness | AOA | PercentageID | 95-90 | 3.92E-05 |
| Richness | AOA | PercentageID | 97-90 | 0 |
| Richness | AOA | PercentageID | ASV-90 | 0 |
| Richness | AOA | PercentageID | 97-95 | 3.80E-08 |
| Richness | AOA | PercentageID | ASV-95 | 0 |
| Richness | AOA | PercentageID | ASV-97 | 0 |
| Richness | AOA | Type | Runnel-Ridge | 0.92289543 |
| Shannon | AOA | PercentageID | 90-85 | 0 |
| Shannon | AOA | PercentageID | 95-85 | 0 |
| Shannon | AOA | PercentageID | 97-85 | 0 |
| Shannon | AOA | PercentageID | ASV-85 | 0 |
| Shannon | AOA | PercentageID | 95-90 | 0 |
| Shannon | AOA | PercentageID | 97-90 | 0 |
| Shannon | AOA | PercentageID | ASV-90 | 0 |
| Shannon | AOA | PercentageID | 97-95 | 1.74E-09 |
| Shannon | AOA | PercentageID | ASV-95 | 0 |
| Shannon | AOA | PercentageID | ASV-97 | 0.99813428 |
| Shannon | AOA | Type | Runnel-Ridge | 0.32124845 |
| Simpson | AOA | PercentageID | 90-85 | 0 |
| Simpson | AOA | PercentageID | 95-85 | 0 |
| Simpson | AOA | PercentageID | 97-85 | 0 |
| Simpson | AOA | PercentageID | ASV-85 | 0 |
| Simpson | AOA | PercentageID | 95-90 | 0 |
| Simpson | AOA | PercentageID | 97-90 | 0 |
| Simpson | AOA | PercentageID | ASV-90 | 0 |
| Simpson | AOA | PercentageID | 97-95 | 0.0560966 |
| Simpson | AOA | PercentageID | ASV-95 | 0.99908246 |
| Simpson | AOA | PercentageID | ASV-97 | 0.08346504 |
| Simpson | AOA | Type | Runnel-Ridge | 0.82231714 |
| Richness | nxrB | PercentageID | ASV-97 | 0.00027696 |
| Richness | nxrB | Type | Runnel-Ridge | 0.61014694 |
| Shannon | nxrB | PercentageID | ASV-97 | 2.10E-14 |
| Shannon | nxrB | Type | Runnel-Ridge | 0.86867832 |
| Simpson | nxrB | PercentageID | ASV-97 | 2.10E-14 |
| Simpson | nxrB | Type | Runnel-Ridge | 0.88343885 |
| Richness | nirK | PercentageID | 90-83 | 0.0001495 |
| Richness | nirK | PercentageID | 95-83 | 0 |
| Richness | nirK | PercentageID | 97-83 | 0 |
| Richness | nirK | PercentageID | ASV-83 | 0 |
| Richness | nirK | PercentageID | 95-90 | 0.00202056 |
| Richness | nirK | PercentageID | 97-90 | 3.52E-07 |
| Richness | nirK | PercentageID | ASV-90 | 0 |
| Richness | nirK | PercentageID | 97-95 | 0.17855445 |
| Richness | nirK | PercentageID | ASV-95 | 0 |
| Richness | nirK | PercentageID | ASV-97 | 7.77E-11 |
| Richness | nirK | Type | Runnel-Ridge | 0.21301092 |
| Shannon | nirK | PercentageID | 90-83 | 0.09841584 |
| Shannon | nirK | PercentageID | 95-83 | 0.00290341 |
| Shannon | nirK | PercentageID | 97-83 | 0.00015804 |
| Shannon | nirK | PercentageID | ASV-83 | 4.68E-11 |
| Shannon | nirK | PercentageID | 95-90 | 0.72285622 |
| Shannon | nirK | PercentageID | 97-90 | 0.24140844 |
| Shannon | nirK | PercentageID | ASV-90 | 3.30E-06 |
| Shannon | nirK | PercentageID | 97-95 | 0.92201595 |
| Shannon | nirK | PercentageID | ASV-95 | 0.00042211 |
| Shannon | nirK | PercentageID | ASV-97 | 0.00682669 |
| Shannon | nirK | Type | Runnel-Ridge | 0.00128093 |
| Simpson | nirK | PercentageID | 90-83 | 0.92544577 |
| Simpson | nirK | PercentageID | 95-83 | 0.86024702 |
| Simpson | nirK | PercentageID | 97-83 | 0.7621883 |
| Simpson | nirK | PercentageID | ASV-83 | 0.0992128 |
| Simpson | nirK | PercentageID | 95-90 | 0.99979552 |
| Simpson | nirK | PercentageID | 97-90 | 0.99607778 |
| Simpson | nirK | PercentageID | ASV-90 | 0.4442688 |
| Simpson | nirK | PercentageID | 97-95 | 0.99968001 |
| Simpson | nirK | PercentageID | ASV-95 | 0.55285908 |
| Simpson | nirK | PercentageID | ASV-97 | 0.6758093 |
| Simpson | nirK | Type | Runnel-Ridge | 0.00185014 |
| Richness | nirS | PercentageID | 90-82 | 0 |
| Richness | nirS | PercentageID | 95-82 | 0 |
| Richness | nirS | PercentageID | 97-82 | 0 |
| Richness | nirS | PercentageID | ASV-82 | 0 |
| Richness | nirS | PercentageID | 95-90 | 0.00700387 |
| Richness | nirS | PercentageID | 97-90 | 1.17E-07 |
| Richness | nirS | PercentageID | ASV-90 | 0 |
| Richness | nirS | PercentageID | 97-95 | 0.03311456 |
| Richness | nirS | PercentageID | ASV-95 | 0 |
| Richness | nirS | PercentageID | ASV-97 | 0 |
| Richness | nirS | Type | Runnel-Ridge | 0.81668163 |
| Shannon | nirS | PercentageID | 90-82 | 0 |
| Shannon | nirS | PercentageID | 95-82 | 0 |
| Shannon | nirS | PercentageID | 97-82 | 0 |
| Shannon | nirS | PercentageID | ASV-82 | 0 |
| Shannon | nirS | PercentageID | 95-90 | 0 |
| Shannon | nirS | PercentageID | 97-90 | 0 |
| Shannon | nirS | PercentageID | ASV-90 | 0 |
| Shannon | nirS | PercentageID | 97-95 | 9.18E-05 |
| Shannon | nirS | PercentageID | ASV-95 | 0 |
| Shannon | nirS | PercentageID | ASV-97 | 0 |
| Shannon | nirS | Type | Runnel-Ridge | 0.40632033 |
| Simpson | nirS | PercentageID | 90-82 | 0 |
| Simpson | nirS | PercentageID | 95-82 | 0 |
| Simpson | nirS | PercentageID | 97-82 | 0 |
| Simpson | nirS | PercentageID | ASV-82 | 0 |
| Simpson | nirS | PercentageID | 95-90 | 0 |
| Simpson | nirS | PercentageID | 97-90 | 0 |
| Simpson | nirS | PercentageID | ASV-90 | 0 |
| Simpson | nirS | PercentageID | 97-95 | 0.10677728 |
| Simpson | nirS | PercentageID | ASV-95 | 0 |
| Simpson | nirS | PercentageID | ASV-97 | 0 |
| Simpson | nirS | Type | Runnel-Ridge | 0.13475708 |
| Richness | nrfA | PercentageID | ASV-97 | 1.00E-13 |
| Richness | nrfA | Type | Runnel-Ridge | 0.02628762 |
| Shannon | nrfA | PercentageID | ASV-97 | 1.00E-13 |
| Shannon | nrfA | Type | Runnel-Ridge | 0.51375652 |
| Simpson | nrfA | PercentageID | ASV-97 | 1.00E-13 |
| Simpson | nrfA | Type | Runnel-Ridge | 0.0037615 |
